# Multiple copies of the oxytetracycline gene cluster in selected *Streptomyces rimosus* strains can provide significantly increased titers

**DOI:** 10.1186/s12934-021-01522-5

**Published:** 2021-02-17

**Authors:** Špela Pikl, Andrés Felipe Carrillo Rincón, Lucija Slemc, Dušan Goranovič, Martina Avbelj, Krešimir Gjuračić, Hilda Sucipto, Katja Stare, Špela Baebler, Martin Šala, Meijin Guo, Andriy Luzhetskyy, Hrvoje Petković, Vasilka Magdevska

**Affiliations:** 1 Department of Food Science and Technology, Biotechnical Faculty, University of Ljubljana, Ljubljana, Slovenia; 2 Acies Bio, d.o.o, Tehnološki Park, Ljubljana, Slovenia; 3 Pharmazeutische Biotechnologie, Universität des Saarlandes, Saarbrücken, Germany; 4 Helmholtz-Institut für Pharmazeutische Forschung Saarland, Saarbrücken, Germany; 5 National Institute of Biology, Večna pot 111, Ljubljana, Slovenia; 6 National Institute of Chemistry, Hajdrihova 19, SI-1000 Ljubljana, Slovenia; 7 State Key Laboratory of Bioreactor Engineering, East China University of Science and Technology, Shanghai, China

**Keywords:** *Streptomyces rimosus*, Oxytetracycline, ΦC31, Biosynthesis, Biosynthetic gene cluster

## Abstract

**Background:**

Natural products are a valuable source of biologically active compounds that have applications in medicine and agriculture. One disadvantage with natural products is the slow, time-consuming strain improvement regimes that are necessary to ensure sufficient quantities of target compounds for commercial production. Although great efforts have been invested in strain selection methods, many of these technologies have not been improved in decades, which might pose a serious threat to the economic and industrial viability of such important bioprocesses.

**Results:**

In recent years, introduction of extra copies of an entire biosynthetic pathway that encodes a target product in a single microbial host has become a technically feasible approach. However, this often results in minor to moderate increases in target titers. Strain stability and process reproducibility are the other critical factors in the industrial setting. Industrial *Streptomyces rimosus* strains for production of oxytetracycline are one of the most economically efficient strains ever developed, and thus these represent a very good industrial case. To evaluate the applicability of amplification of an entire gene cluster in a single host strain, we developed and evaluated various gene tools to introduce multiple copies of the entire oxytetracycline gene cluster into three different *Streptomyces rimosus* strains: wild-type, and medium and high oxytetracycline-producing strains. We evaluated the production levels of these engineered *S. rimosus* strains with extra copies of the oxytetracycline gene cluster and their stability, and the oxytetracycline gene cluster expression profiles; we also identified the chromosomal integration sites.

**Conclusions:**

This study shows that stable and reproducible increases in target secondary metabolite titers can be achieved in wild-type and in high oxytetracycline-producing strains, which always reflects the metabolic background of each independent *S. rimosus* strain. Although this approach is technically very demanding and requires systematic effort, when combined with modern strain selection methods, it might constitute a very valuable approach in industrial process development.

## Background

Secondary metabolites produced by diverse microorganisms represent a very important source of medical and agricultural products [1, 2]. However, a clear limitation of natural products is the relatively slow and work-intensive strain improvement and process development that has to be carried out to ensure that commercially produced target metabolites meet the necessary quality requirements [3, 4]. Due to the complexity of metabolic pathways, including complex regulation networks for secondary metabolite biosynthesis, industrial strain improvements to increase titers still predominantly rely on technically advanced random mutagenesis and strain selection regimes [5]. In addition to advanced strain selection methods, modern metabolic engineering approaches are nowadays indispensable tools in industrial strain and process development. Although significant efforts have been invested in strain selection methods, many industrial bioprocesses have not been significantly improved in decades, and the conversion of a carbon source to the target product does not generally exceed 10 %. This is the case for high-titer industrial strains, and particularly for those related to mature products. This thus poses a serious threat to the economic and industrial viability of these processes. On the other hand, when considering, for example, the production of primary metabolites, the conversion rate can often reach 50 % [6].

In metabolic engineering approaches, modulation of the expression of genes or pathways involved in the regulation of secondary metabolite biosynthesis is an approach that can be productive in the early stages of strain improvement regimes [7]. Increasing the substrate supply of a primary metabolite to ensure a supply of building blocks to be used for the biosynthesis of target secondary metabolites is often a very productive approach in strain improvement strategies [8]. However, when applying these approaches to advanced industrial strains, which already produce very high titers of their target products, they seldom result in further titer increases [5]. Augmenting ‘gene dosage’ by amplification of the target gene or the entire biosynthetic pathway might instead be a successful strategy [9]. This can be achieved by expression of the target biosynthetic pathway on a replicative plasmid, which is maintained in the host in multiple copies [10]. Alternatively, multiple copies of genes that encode the biosynthesis of a target product can be integrated into the chromosomes [11]. Both approaches have their advantages and disadvantages regarding efficacy, reproducibility, and strain stability. One of the most successful examples of this strategy is the amplification of the *rib*-operon in the biosynthesis of vitamin B2, where the introduction of multiple copies of the rib-operon in a replicative plasmid along with the chromosomally integrated copies resulted in very significant titer increases [12].

Interestingly, spontaneous amplification of an entire biosynthetic pathway that encodes secondary metabolite biosynthesis can occur during strain improvement regimes [11]. This has been observed with large biosynthetic gene clusters (BGCs) that encode secondary metabolite pathways, as seen for example in some industrial *Penicillium chrysogenum* strains that are high producers of penicillin [13], where the penicillin titer increases with the number of copies of the penicillin BGC. However, spontaneous amplification of entire BGCs that encode the biosynthesis of complex secondary metabolites is generally not observed in industrial high-producing strains during strain-improvement campaigns, with some exceptions [11].

In recent years, metabolic engineering has become a powerful strategy to optimize secondary metabolism in actinomycetes [14], and the introduction of extra copies of entire BGCs has become a technically feasible approach [15]. A number of successful attempts to introduce additional copies of BGCs into producing actinomycete strains have been carried out, where additional chromosomally integrated copies of entire BGCs have resulted in titer increases [15–17]. Generally, nonindustrial actinomyces strains have been used, with relatively minor increases in titers. However, Manderscheid et al. (2016) demonstrated that multigram concentrations of target product per liter of culture can be achieved, thus showing that the introduction of additional copies of BGCs can constitute a productive approach to increase titers of secondary metabolites [15]. However, secondary metabolites are most often produced on a large scale, as multi-thousands of tons per year, and thus extremely high titers of the target product are necessary to achieve sufficient supply and to meet economic demands [5]. In addition to a high titer, some of the most important properties of the industrial high OTC-producing strains are also the stability and reproducibility of the industrial strains. Thus, when considering an industrial application, it is important to evaluate novel biosynthetic engineering approaches for the current industrial super-producing strains.

*Streptomyces rimosus* OTC-producing strains are some of the most economically efficient industrial strains ever developed, with the best of these producing > 30 g/L OTC [18]. In this study, we selected three *S. rimosus* strains, here designated as the wild-type *S. rimosus* ATCC 10970 strain; the medium OTC-producing M4018 strain; and the high OTC-producing HP0508 strain. We developed and evaluated efficient gene tools that allowed us to introduce multiple copies of an entire *otc* gene cluster into all three of these *S. rimosus* strains. We evaluated their OTC production and selected *otc* gene expression profiles. Importantly, we identified the exact chromosomal integration sites, and evaluated the stability of the strains with significant titer increases. This study shows that titer increases of this target secondary metabolite, i.e., OTC, can be achieved in industrial strains. However, this is not always the case, as this depends on the specific strain, which appears to reflect the metabolic background of each strain that is developed during intensive strain-selection regimes.

## Results

### Cloning of the entire otc gene cluster from *S. rimosus* through application of a single-step transformation-associated recombination approach

The DNA fragment of 31,079 bp in length that contains the entire *otc* gene cluster (Additional file 1: Figure S1) was cloned directly from genomic DNA of the *S. rimosus* high OTC-producing HP0508 strain, using transformation-associated recombination (TAR) cloning in *S. cerevisiae* [19]. The partial restriction of chromosomal DNA isolated from *S. rimosus* with the *Spe*I restriction enzyme stimulated a double-crossover recombination event between the plasmid bearing linear homologies and the chromosomal DNA, thus allowing assembly of the *otc* gene cluster in a YAC plasmid. The presence of the entire *otc* cluster cloned into pYAC-ΦC31-Ts was initially confirmed in selected yeast transformants by colony PCR screening. Five of 150 transformants screened using colony PCR showed positive PCR amplicons with all four sequence-tagged-site primer pairs used, which indicated the presence of an entire *otc* gene cluster in the pYAC-ΦC31-Ts plasmid (data not shown; see Sect. "Cloning of the entire *otc* gene cluster from *S. rimosus* HP0508 by the single-step transformation associated recombination approach".).

Five selected transformants containing the *otc* gene cluster were analyzed by restriction analysis with *Sac*I after retransformation of the plasmid from yeast into *Escherichia coli*. All of the plasmid clones analyzed showed the expected restriction enzyme pattern, and thus contained the entire *otc* gene cluster (Additional file 1: Figure S2). This demonstrated that all five of these individual yeast clones contained the entire *otc* cluster with no evident rearrangements, which confirmed their genetic stability during propagation in both yeast and *E. coli*.

### Construction of *S. rimosus* ΔOTC mutants

It was important to evaluate the functionality of the constructed integrated pYAC-ΦC31-Ts-OTC plasmid that contained the entire *otc* gene cluster, and to evaluate the impact of the new location of the chromosomally integrated *otc* gene cluster on the OTC titer. We thus initially deleted the *otc* gene cluster from the *S. rimosus* ATCC 10970 and HP0508 strains. The results of the complementation of these *S. rimosus* deletion strains are given in Sect. "Complementation of *S. rimosus* ATCC 10970ΔOTC, 15883S, and HP0508ΔOTC deletion strains by ΦC31 phage integrase-mediated chromosomal integration of the *otc* gene cluster".

Here, different approaches were used for deletion of the *otc* gene cluster; however, these resulted in almost identical gene deletion events in the ATCC 10970 and HP0508 strains. The *S. rimosus* 15883S strain is a spontaneous mutant of the M4018 progenitor strain, with a deleted *otc* cluster [20], where the deletion resulted in removal of the entire *otc* BGC and its surrounding region [20]. The iterative marker excision system was used for the ATCC 10970 strain (Sect. "Deletion of the otc cluster in* S. rimosus* ATCC 10970"], and homologous recombination was used for the HP0508 strain (Sect. "Deletion of the otc gene cluster in* S. rimosus* HP0508".]. Both of these methods resulted in a large deletion (approximately 21,000 bp) of the internal part of the *otc* gene cluster. Genes responsible for resistance (*otrB*) and regulation (*otcR, oxyTA1, otcG*) remained in the chromosome of ATCC 10970, and in addition to these genes, the resistance gene *otrA* remained in the HP0508 strain (Additional file 1: Figure S1).

After sub-cultivation, the colonies were patched on the soya-mannitol agar (MS) medium without and with selective pressure, until the colonies sensitive to thiostrepton were identified. Colonies sensitive to thiostrepton had the deletion of *otc* cluster confirmed by colony PCR (data not shown). All of the deletion mutants had the ‘white’ phenotype, as opposed to the light brown phenotype with white spores of the parental strain colonies of all three strains (Additional file 1: Figure S3). The deletion mutants were further tested by cultivation in a production medium, as described in Sect. "Media and culture conditions", and their production of OTC was examined. HPLC analysis confirmed that none of the three ΔOTC mutants produced OTC (Additional file 1: Figure S4).

It is also important to emphasize that the identical production medium (GOTC-P; Sect. "Media and culture conditions".) was used for all three strains under evaluation, which is not an optimal medium for any of them; all of the *S. rimosus* industrial media are based on starch as the main carbon source, and therefore GOTC-P medium was used for the comparative study of all three of these strains. As expected, the OTC titers for the ATCC 10970 and M4018 strains (~ 100 mg/L, ~ 3 g/L OTC, respectively), were lower than that of the high OTC-producing HP0508 strain (~ 8 g/L OTC) (Additional file 1: Figure S4).

### Complementation of* S. rimosus* ATCC 10970ΔOTC, 15883S, and HP0508ΔOTC deletion strains by ΦC31 phage integrase-mediated chromosomal integration of the *otc* gene cluster

Three different *S. rimosus* ΔOTC strains were transformed with the pYAC-ΦC31-Ts-OTC plasmid that contained the entire *otc* gene cluster. The transformants were selected on MS medium with selective pressure to ensure incorporation of the plasmid into the ΔOTC genome. Independent transformants of three different ΔOTC strains that putatively carried the *otc* gene cluster integrated into the chromosome were tested for OTC production (Sects. "Media and culture conditions" and "High performance liquid chromatography"). The OTC titers of independent morphologically stable transformants were determined after 5 days of cultivation in 5-mL cultures, compared to the control nontransformed ΔOTC strains and the parental strains. As an additional control, we have also transformed all three parental strains with empty plasmid pYAC-ΦC31-Ts-h (i.e., without the *otc* cluster).

Most of the independent transformants of the M4018 and HP0508 deletion strains showed similar OTC titers when compared to their M4018 and HP0508 parental strains, respectively. The transformants of the ATCC 10970 deletion strain even showed an increase in OTC titer when compared to the ATCC 10970 parental strain, thus confirming that the cloned *otc* gene cluster was fully functional (Additional file 1: Figure S5). We observed minor, but statistically significant increase in OTC titer when M4018 and HP0508 parental strains were transformed with the empty plasmid pYAC-ΦC31-Ts-h (Additional file 1: Figure S6, See Sect. "Introduction of additional copies of the *otc* gene cluster in the *S. rimosus* ATCC 10970, M4018, and HP0508 strains"). However, when transforming ATCC 10970 parental strain with the empty pYAC-ΦC31-Ts-h plasmid, we did not observe statistically significant increase of the OTC titer (Additional file 1: Figure S6).

**Fig. 1:**
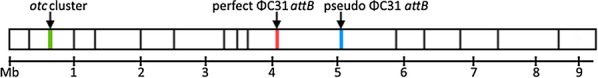
Proposed map of the *S. rimosus* M4018 chromosome. The vertical black lines represent the *AseI* restriction sites. The horizontal line below the map indicates where the expected positions are in the *S. rimosus* chromosome (in Mb), based on the entire genome of the ATCC 10970 strain. The red and blue lines indicate the surrounding sequences of the ΦC31integration sites identified by plasmid-rescue and PFGE. The green line shows the location of the native *otc* BGC

Interestingly, although the *otc* gene cluster in the ATCC 10970 was relocated into the core region of the chromosome (~ 600 kb from one end of the chromosome; Fig. 1), the OTC titer produced by the engineered strains was not reduced. On the contrary, the OTC titer for many of the independent transformants was increased. This effect was most prominent in the ATCC 10970 strain, where the OTC titer was significantly increased, with many of the transformants showing up to 5.5-fold increases (> 1 g/L OTC), compared to the parental strain (≤ 200 mg/L OTC); this was indeed unexpected. Following complementation with the *otc* BGC, the morphology of the transformants returned from the white phenotype in the *otc*-deleted strains to the light brown mycelium with white spores, as comparable to the parental strains. Complementation of the ATCC 10970, M4018, and HP0508 deletion mutants with the pYAC-ΦC31-Ts-h plasmid did not result in restoration of OTC production, nor did it have any impact on any other phenotype. The location of the chromosomal integration sites were further analysed in Sect. "Identification of the pYAC-ΦC31-Ts plasmid integration site in the chromosome of the *Streptomyces rimosus *M4018 strain"

### Introduction of additional copies of the *otc* gene cluster in the *S. rimosus* ATCC 10970, M4018, and HP0508 strains

**Fig. 2:**
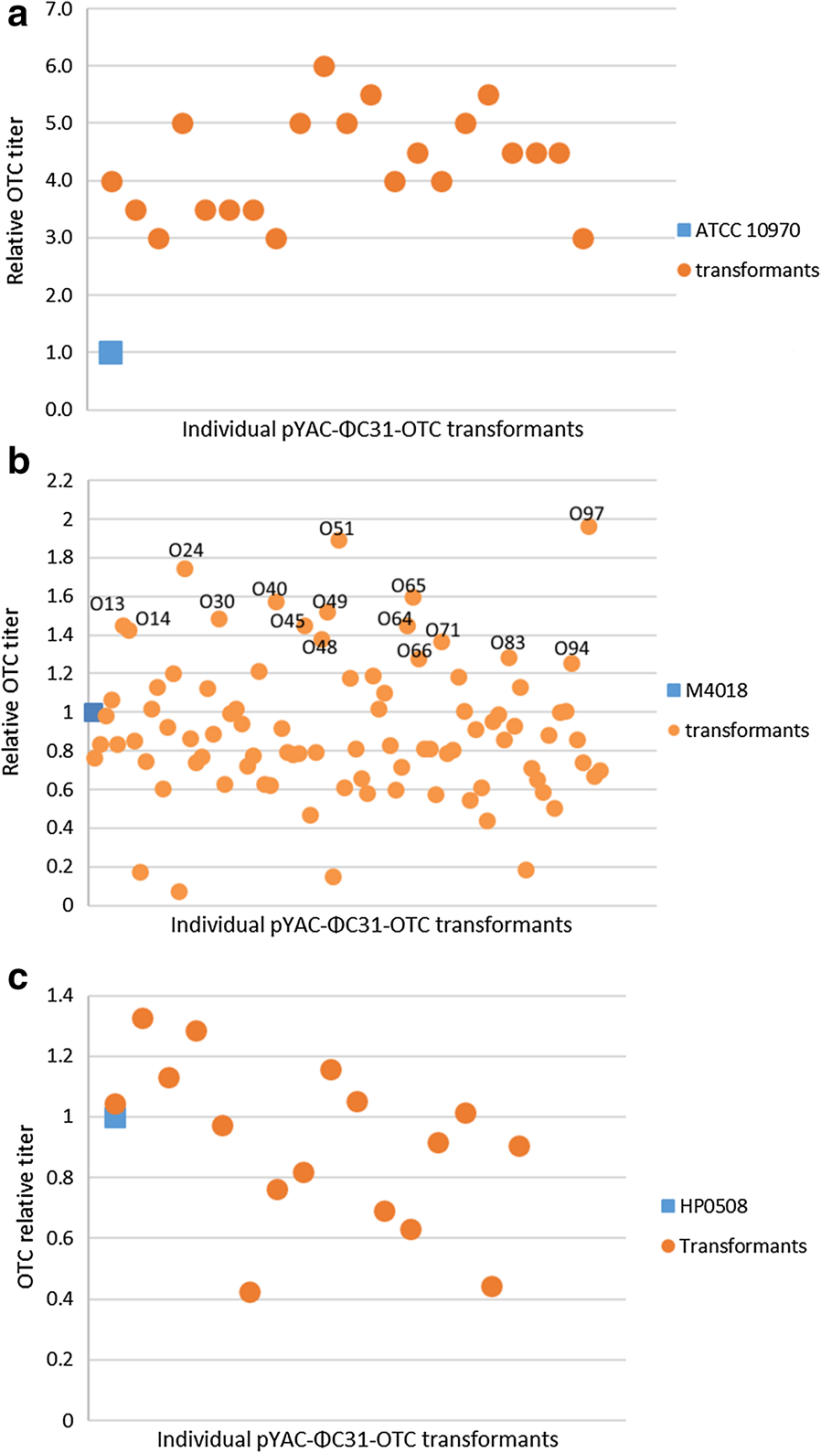
Relative oxytetracycline (OTC) titers of the selected transformants of *S. rimosus* ATCC 10970, M4018 (medium OTC producer), and HP0508 (high OTC producer) parental strains after 5 days of fermentation in 5 mL GOTC production medium. **a** The 21 independent *S. rimosus* ATCC 10970::otc transformants (circles) compared to the *S. rimosus* ATCC 10970 parental strain (square). **b** The 90 independent *S. rimosus* M4018::otc transformants (circles) compared to the S. *rimosus* medium OTC-producing M4018 industrial parental strain (square). The 16 highest producing independent transformants are indicated. **c** The 16 independent *S. rimosus* HP0508::otc transformants (circles) compared to the *S. rimosus* high OTC-producing HP0508 parental strain (square). Each of the independent transformants were analyzed in duplicate; parental strains were cultivated in triplicate; means of OTC titers are shown

Considering that complementation of all three strains lacking the *otc* gene cluster did not have any negative effects on the OTC titer, which demonstrates that the pYAC-ΦC31-Ts-OTC plasmid was fully functional, we proceeded with experiments where additional copies of the *otc* gene cluster was introduced into the ATCC 10970, M4018, and HP0508 strains. Morphologically stable transformants were selected by patching on MS plates supplemented with thiostrepton. The transformants were tested for their production of OTC, in comparison with the nontransformed ATCC 10970, M4018, and HP0508 parental strains. These data demonstrated a significant increase in OTC production for the ATCC 10970 strain. Although many independent transformants resulted in higher OTC titers compared to the control strains, this was observed more rarely in the M4018 and HP0508 strains (Fig. 2). All of the ATCC 10970 transformants showed an increase in OTC titers. Remarkably, compared to the parental strain, the majority of ATCC 10970 transformants produced two-fold to five-fold higher titers, and reached > 1 g/L OTC at the end of the fermentation (Fig. 2). In contrast to the ATCC 10970 strain transformants, only relatively moderate increases in OTC titer were obtained for some of the M4018 and HP0508 transformants. Compared to the M4018 parental strain (~ 3 g/L OTC), a large number of the M4018 transformants reached absolute OTC titers of > 6 g/L OTC, thus with twice the parental OTC titer, and approaching the titer of the high OTC-producing HP0508 strain (Fig. 2b). Therefore, a larger number of independent transformants were tested, with the final selection of 16 morphologically stable M4018 transformants for further study (Fig. 2b, numbered). Some independent HP0508 transformants achieved higher titers of OTC compared to the HP0508 parental strain (Fig. 2c), with some up to 1.4-fold higher. This represents a particularly high increase, considering that these were derived from a high OTC-producing strain with generally ~ 8 g/L OTC following fermentation in a shaking flask.

The empty pYAC-ΦC31-Ts-h plasmid (i.e., without the *otc* cluster) was used as a control here. Of note, the transformants obtained with the control plasmid also showed some variability in their OTC titers. This was seen in particular for the M4018 and HP0508 strains, where the variability was significant (P < 0.05; Mann-Whitney tests). Thus, transformation of the M4018 and HP0508 strains with the empty plasmid resulted in some moderate increases in the OTC titer. In contrast, the variability was low in the ATCC 10970 strain (P > 0.05), with no significant changes in OTC production (Additional file 1: Figure S6). We then continued with this study with the 16 M4018::OTC mutants that had the highest OTC titers.

### Reproducibility of the OTC titers for the *S. rimosus* M4018 strain containing multiple copies of the *otc* gene cluster

**Fig. 3:**
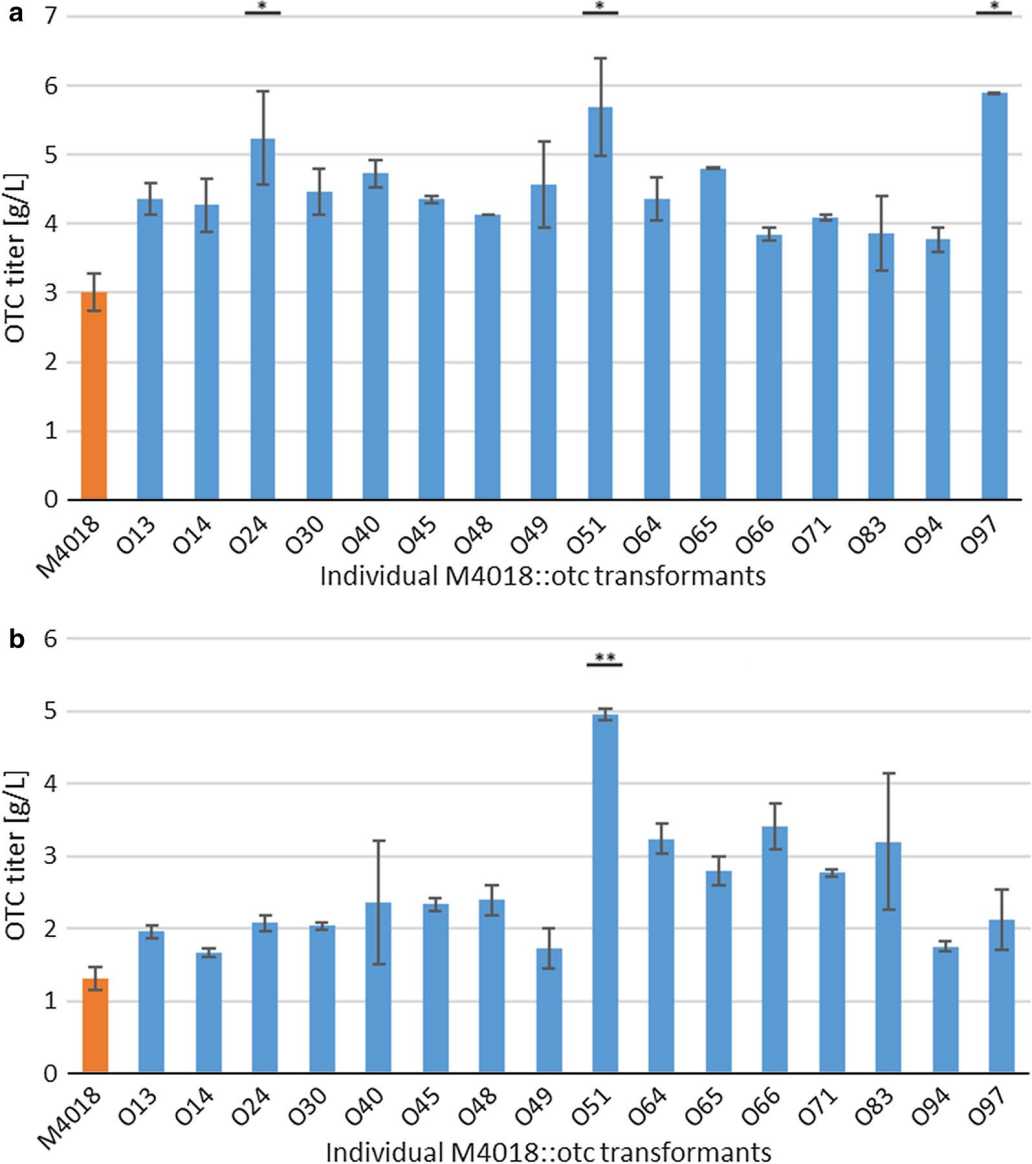
Oxytetracycline (OTC) titers of the 16 best independent *S. rimosus* M4018::otc transformants, as achieved after 5 days of cultivation. Testing of the transformants was carried out in duplicate. **a** First re-testing on falcon tube scale. **b** Second re-testing on 250 mL glass flask scale. * P < 0.05; **P < 0.001 (vs. control M4018 parental strain; one-way between-subjects ANOVA)

Re-testing of the selected M4018 transformants was carried out on falcon tube scale and in 250-mL glass flasks in 25 mL GOTC production medium (Fig. 3) and the increases in the OTC titers were monitored. In the second re-test, the OTC titers in all of the M4018 transformants decreased, although also for the M4018 parental strain (control) (Fig. 3). Thus, with these data normalized to the control (i.e., the M4018 parental strain), their relative titers did not decrease. To confirm this, one-way between-subjects ANOVA was used to compare the effects of multiple *otc* BGC copies on OTC titers (SPSS Statistics for Windows; 21). Here, 16 of the tranformants showed significant increases in OTC production compared to the M4018 parental strain. Across the first tests, the three O24, O51 and O97 transformants showed the highest significant responses for the OTC titers, as 1.7-fold to 2.0-fold the M4018 parental strain. These three showed significant effects of the additional *otc* BGC copies over the M4018 OTC titer at the p < 0.05 level [F(16, 17) = 3.842; p = 0.004]. *Post-hoc* comparisons using Tukey HSD tests indicated that the mean scores for the O24, O51 and O97 transformants were indeed significantly greater compared to the M4018 parental strain for the first re-test (Fig. 3a). However, in the second re-test, only the O51 transformant showed a significantly greater response over the M4018 parental strain (Fig. 3b). These three transformants were selected for further studies, together with three other transformants, O45, O64 and O83, with relative OTC titer responses of 1.3-fold to 1.4-fold.

### Genome analysis of selected *S. rimosus* M4018 transformants with significantly higher OTC titers

To identify the number of copies incorporated into the genomes of these selected M4018 transformants, O24, O45, O51, O64, O83 and O97, their total DNA was isolated in agarose plugs and digested with either *Xho*I or the rare-cutter enzyme *Ase*I. Chromosomal DNA from the nontransformed M4018 parental strain and a randomly selected transformant obtained with the empty pYAC-ΦC31-Ts-h plasmid (O4) were used as the controls.

**Fig. 4:**
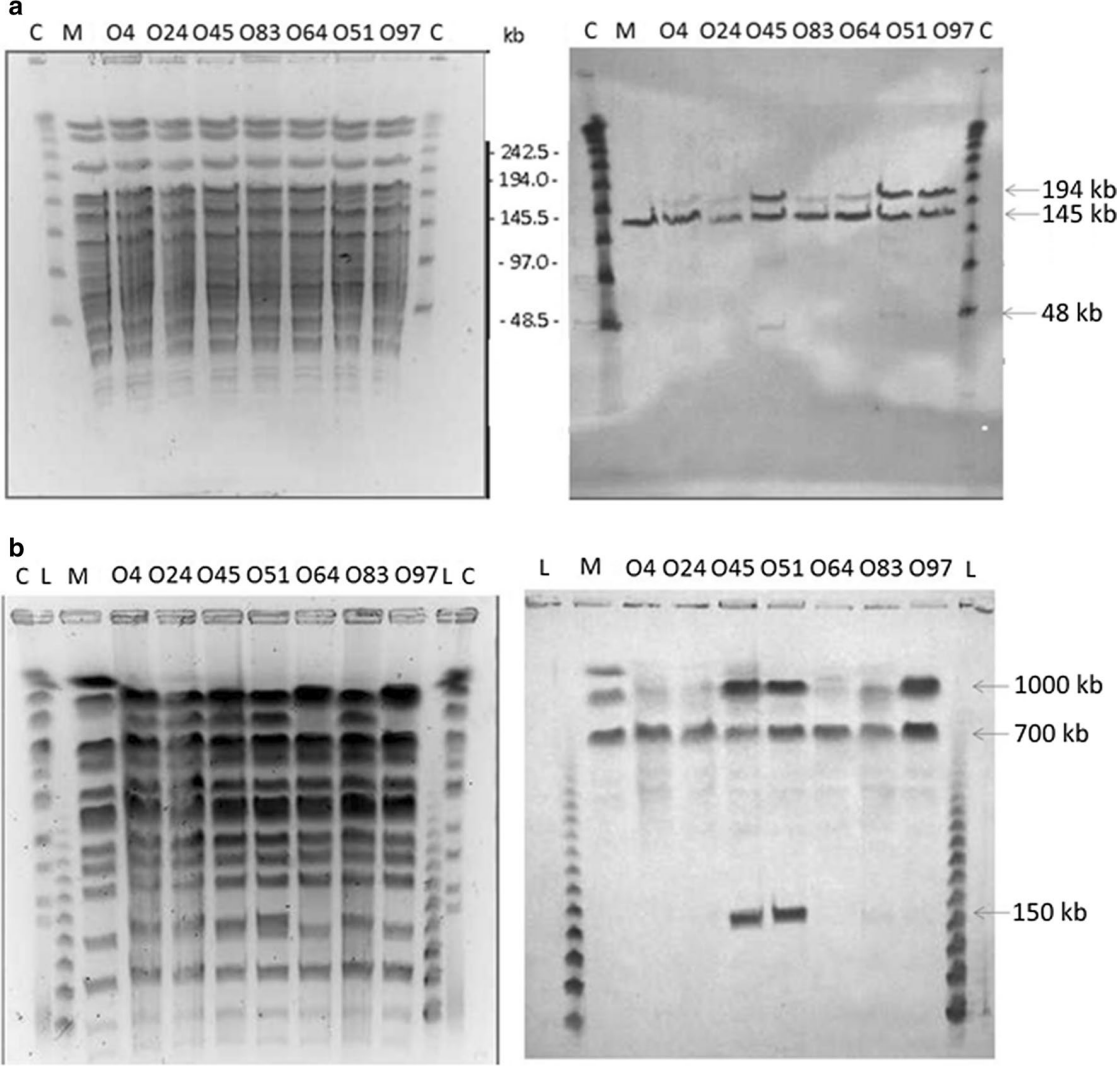
Pulse-field gel electrophoresis (PFGE; left) and Southern blotting (right) of *S. rimosus* M4018 transformants (as indicated) carrying the chromosomally integrated pYAC-ΦC31-Ts-OTC plasmid. **a** Genomic DNA digested with the *Xho*I restriction enzyme. **b** Genomic DNA digested with the *Ase*I restriction enzyme. The Southern blotting (right) of the PFGE gels (left) was through hybridization with probes generated from external homologies of pYAC ΦC31-Ts-h. L, Lambda concatamers, C, *S. cerevisiae* chromosomes; M, *S. rimosus* M4018 parental strain; O4, *S. rimosus* M4018 transformant with pYAC-ΦC31-Ts-h (control); O24-O97, selected *S. rimosus* M4018::otc transformants. Arrows indicate presence of the *otc* cluster

Pulse-field gel electrophoresis (PFGE) was carried out, and these gels were used for Southern hybridization with digoxigenin (DIG)-labeled DNA probes prepared from DNA fragments of external homologies of the *otc* gene cluster located on the pYAC-ΦC31-Ts-h plasmid (Additional file 1: Figure S1, Fig. 4). All of the transformants analyzed showed at least one additional band when compared to the *S. rimosus* M4018 parental strain, which indicated at least one additional copy of the *otc* gene cluster integrated chromosomally in another position on the M4018 chromosome (Fig. 4). The O45 and O51 transformants showed more than one additional band when compared to the M4018 parental strain, thus indicating that multiple copies of the *otc* gene cluster were introduced during the conjugation. Interestingly, these O45 and O51 transformants were two of the best-performing M4018 transformants (Fig. 3). Considering that multiple copies of the *otc* gene cluster can result in additional instability of such engineered strains, we carried out additional strain stability tests.

### Genetic stability of *S. rimosus* M4018 strains containing extra copies of the *otc* gene cluster

We evaluated the genetic stability of the O51 transformant, that had extra copies of the *otc* gene cluster, with the M4018 transformant with the pYAC- ΦC31-Ts-h plasmid (O4) and the M4018 parental strain as controls. Genetic stability was tested using two different techniques: measurement of the OTC titers in the absence and presence of a resistance marker; and by PFGE and Southern blotting, as described in Sects. "Pulse-field gel electrophoresis" and "Probe design and Southern blotting".

It is important to maintain this stability during seed culture cultivation, the degree of strain stability was evaluated in terms of the absence and presence of thiostrepton in the seed cultures. This seed was then used as inoculum for the production medium, as described in Methods, Sect. "Media and culture conditions". The OTC titers were then analyzed after 5 days of cultivation. As the O51 transformant had shown the highest OTC titers, it was selected for this analysis. Therefore, the O51 colony and seven colonies derived from it were compared to the M4018::YAC-h transformant O4 and the M4018 parental strain (Additional file 1: Figure S7). Mann-Whitney tests were carried out to determine whether the thiostrepton in the medium had any significant influence on the OTC production of these selected colonies. This showed that the OTC titers were significantly enhanced without and with thiostrepton in the medium, in comparison to the O4 control. The colonies cultivated in the seed medium with thiostrepton resulted in more reproducible OTC production at slightly higher OTC titers (Additional file 1: Figure S7a). We can thus conclude that the OTC production in the absence of thiostrepton is less reproducible, and that the OTC titers are lower; however, the OTC titers were still significantly enhanced. It is known that microbial cultures grow faster without antibiotic pressure, and therefore seed cultures mature much faster. Considering that the control strains also showed significantly lower OTC titers here, this most likely resulted from nonoptimal seed quality (Additional file 1: Figure S7b).

The second analysis of the genome stability in the transformants with the additional integrated copies of the *otc* gene cluster was by PFGE and Southern blotting. The O51 transformant was again selected here, which based on the data shown above (Sect. "Genome analysis of selected *S.rimosus* M4018 transformants with significantly higher OTC titers".), contained two additional copies of the *otc* gene cluster in its genome. Seven O51-derived colonies were cultivated in 5 mL TSB medium for 24 h without thiostrepton, and the genomic DNA was then extracted and digested with the *Ase*I or *Xho*I restriction enzymes. Following the PFGE and Southern blotting, all of the replicates except O51-4 showed high genetic stability, as both of the additional bands from the *otc* gene clusters remained inserted in the genome (Additional file 1: Figure S8). Accordingly, the titer of OTC of colony O51-4 is reduced on the medium without thiostrepton (Additional file 1: Figure S7B).

### **Identification of the pYAC-ΦC31-Ts plasmid integration site in the chromosome of the*Streptomyces rimosus*M4018 strain**

We aimed next to identify the location of the chromosomal integration of the ΦC31 recombinase-based pYAC-ΦC31-Ts plasmid in these transformants (Additional file 1: Figure S9). It was reasonable to expect that these M4018 transformants carrying the pYAC-ΦC31-Ts plasmid should have the exogenous DNA integrated in the perfect *attB* site. This will introduce two additional *Ase*I restriction sites into these *S. rimosus* genomes with each integration event, and thus change the restriction patterns of the M4018 genome. However, as the two *Ase*I restriction sites are very close together, they can be considered as a single site for this PFGE analysis, and therefore we refer to them as one restriction site. Restriction endonuclease analysis was carried out with *Ase*I, combined with PFGE of the different *S. rimosus*::YAC transformants. As shown in Additional file 1: Figure S10, three different restriction patterns were observed following the PFGE electrophoresis, which thus suggested multiple *attB* sites specific for ΦC31 in the *S. rimosus* genome.

As the M4018 genome has not been assembled, we used PFGE analysis and compared this to the published genome of ATTC 10970 (NCBI GenBank Accession number: NZ_CP023688) as the reference for this analysis of the M4018 chromosome. Based on this ATCC 10970 genome sequence, some conclusions can be drawn about the *attB* site in the M4018 genome. The ATCC 10979 genome sequence showed 13 *Ase*I restriction sites; however, due to the similar sizes of some of the fragments, these cannot be separated by PFGE, and thus 13 fragments were observed after PFGE instead of 14 expected (Additional file 1: Figure S10). By comparing the PFGE patterns of *S. rimosus* ATCC 10970, 15883S, M4108 parental strain, and the M4018::YAC transformants, two hybridization patterns were observed for the pYAC-ΦC31-Ts transformants, thus confirming that ΦC31 recombinase can integrate once or twice simultaneously into the *S. rimosus* chromosome (Additional file 1: Figure S10b, c). Both of these *attB* sites for the ΦC31 integrase were located in the 2.3 Mb fragment, as indicated by the differences in the hybridization patterns of the control ATCC 10970, 15883S and M4018 strains in comparison to the four pYAC-ΦC31-Ts transformants (Additional file 1: Figure S10b, c, lanes 4, 5, 6, 7). A single integration event of pYAC-ΦC31-Ts in this location introduced an extra *Ase*I into the 2.3-Mb fragment, and hence the *Ase*I restriction pattern was altered. This gave rise to two bands of 1.3 Mb and 1.0 Mb (Additional file 1: Figure S10a, transformant in lane 6). An additional *attB* site was located at ~ 250 kb away from the first *Ase*I restriction site of the 2.3 Mb fragment. Therefore, a double integration event where pYAC-ΦC31-Ts appeared to have incorporated two *Ase*I restriction sites saw cleavage of the 2.3 Mb fragment twice, thus resulting in three bands, of 250 kb, 970 kb, and 1.1 Mb (Additional file 1: Figure S10a, transformant lane 7). These results are schematically represented in Additional file 1: Figure S11. Although the restriction patterns of the pYAC-ΦC31-Ts transformants in lanes 6 and 7 of Additional file 1: Figure S10a were clear, that of the M4018::YAC transformants in lanes 4 and 5 of Additional file 1: Figure S10a remained ambiguous. This suggested that a subpopulation of each transformant had integrated the plasmid vector only once.

To identify the exact location of the *attB* sites in the M4018 genome that were targeted by the ΦC31 recombinase, a pYAC-ΦC31-Ts plasmid-rescue approach was carried out on the *S. rimosus* chromosome. This approach allows the recovery of the plasmid and the flanking genomic sequences [22], thus indicating the identification of the sequences at the *attL* sites following the targeting of the *attB* sites by the ΦC31 recombinase in the M4018::YAC transformants. The pYAC-ΦC31-ts plasmid architecture allowed us to rescue the genomic sequence upstream of the *Sac*I restriction site (Additional file 1: Figure S9), and therefore the rescued plasmids go backwards from the CEN4 origin of replication to the first *Sac*I restriction site that is located in the M4018 genome, including the *attL* sites and the flanking genomic region upstream of the ΦC31 *attB* sites (Additional file 1: Figure S9). Two different restriction patterns were generated when the rescued plasmids were digested with *Bam*HI/*Nco*I (Additional file 1: Figure S12A). Identification of the *attB* sites was possible through sequencing of the rescued plasmids using the ΦC31 fish Rv primer (Additional file 1: Figure S9; Table S1). The DNA sequences obtained with the ΦC31 fish Rv primer using the two different rescued plasmids as templates were located in the *S. rimosus* genome, and the Frame plot software [23] enabled us to identify the open reading frames (ORFs) targeted by the ΦC31 int/attP recombinase.

Thus this plasmid rescue in combination with PFGE and Southern blotting, plus the data related to the ATCC 10970 genome, allowed us to identify the exact location of the ΦC31 *attB* sites. The perfect *attB* site that contained the correct *attB* DNA sequence was within a putative gene that encodes a pirin-like protein. This pirin-like protein lies within the central part of the ATCC 10970 chromosome (Fig. 1). The second integration site revealed an imperfect, so-called pseudo *attB* site, which was located within a putative acyl-CoA dehydrogenase gene, approximately 940 kb from the perfect *attB* site (Fig. 1). Both of these genes that contained the *attB* sites were disrupted when the plasmid DNA was integrated at these locations. Further analysis of the two rescued DNA sequences allowed the identification of the complete sequences of the perfect *attB* and pseudo *attB* sites (Additional file 1: Figure S12b). As expected, both of these *attB* sites contained the core signature sequence of 5´TT, where the crossover occurs (Additional file1: Figure S12b) [24–26].

### RT-qPCR expression analysis of the selected transformants containing multiple copies of the *otc* gene cluster

The M4018 transformants that contained the three copies of the *otc* gene cluster (i.e., native plus two extra copies integrated) reproducibly produced significantly higher OTC titers, the best of which almost reached the titer of the high OTC-producing HP0508 industrial strain. To confirm that the highest titer achieved was due to the multiple integrated copies of the *otc* cluster, and thus presumably caused by higher *otc*-cluster expression, we evaluated gene expression using RT-qPCR analysis. Expression of the *otc* gene cluster in the O51 transformant that contained the two additional copies of the *otc* cluster was analyzed and compared with the M4018 parental strain and the high OTC-producing HP0508 strain. For this, we selected five genes located in the middle and on the extremities of the *otc* gene cluster (Additional file 1: Figure S1). These genes are involved in OTC resistance (efflux pump; *otrB*), regulation (OTC responsive repressor; *otrR*, *oxyTA1*), minimal polyketide putative β-ketoacyl synthase (PKS; *oxyA*), and late stage C5 and C6 hydroxylation (*oxyS*). Each of these five selected genes is the first gene in the corresponding putative operon (Additional file 1: Figure S1).

**Fig. 5:**
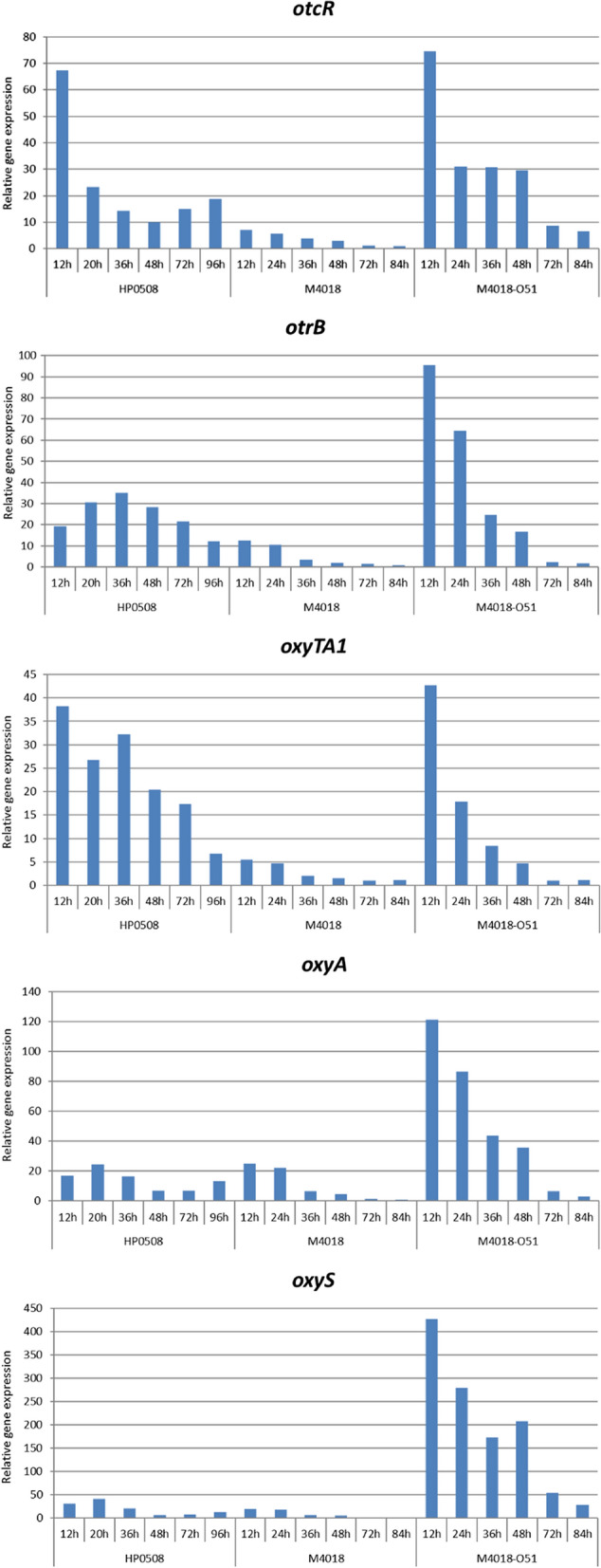
Comparative RT-qPCR analysis of the five selected genes, *otcR* (**a**), o*trB* (**b**), *oxyTA1* (**c**), *oxyA* (**d**), and *oxyS* (**e**), in the three *S. rimosus* strains, as the high OTC-producing HP0508 industrial strain (left), the M4018 parental strain (middle), and the O51 transformant (right). Samples from RT-qPCR were collected at the times indicated following cultivation in GOTC medium. For each gene, the relative gene expression was normalized to 16S rRNA and scaled to the sample with the lowest expression. Full relative expression data is available in Additional file 1: Table S2

These results showed significantly increased transcription profiles of all five of these genes in the O51 transformant, compared to the M4018 parental control, which was apparent at all analysis times (Fig. 5, Additional file 1: Table S2). The most prominent difference was for transcription of the C6 and C5 hydroxylase gene o*xyS* (Additional file 1: Figure S13). Interestingly, the expression levels of the *otrR* and *oxyTA1* genes that are involved in the regulation of the *otc* gene cluster in the HP0508 strain were much higher compared to the M4018 parental strain. However, they showed comparable expression profiles when compared to the O51 transformant, which has three copies of the *otc* gene cluster in the genome; hence the reason for the relatively similar titers in the O51 transformant and HP0508 strain. Interestingly, expression of the *otcR*, *oxyA*, and *oxyS* genes was even stronger in the O51 transformant compared to the HP0508 strain. However, in later stages of the fermentation, the *otc* cluster gene expression lasted much longer in the HP0508 strain compared to the M4018 parental strain and the 051 transformant; this might be the reason for the higher productivity of the HP0508 strain. Transcriptional levels of genes involved in OTC resistance and regulation (*otrB, otrR*) and OTC biosynthesis (minimal PKS *oxyA*; C6/5 hydroxylase *oxyS*) were similar in the HP0508 and M4018 strains. We can therefore conclude that as a result of increased ‘gene dosage’, the expression of all of the genes tested within the scope of this study was significantly increased in the O51 transformant, compared to the M4018 parental strain.

## Discussion

The introduction and stable maintenance of extra copies of target BGCs in actinomycete hosts requires demanding methodology that is often non-productive, as BGCs are highly regulated sequences, and are most often in excess of 50 kb in size. Stability of the integrated BGCs is also an important issue when multiple copies of the target BGCs are present in the chromosome of high OTC-producing strains, as industrial cultures are generally unstable and require important effort for their maintenance [5]. Therefore, within the scope of the present study, we constructed a number of tools and investigated several control conditions to better understand the impact of the strain background and what engineering efforts could be applied.

We constructed an integrative plasmid that carries the entire *otc* BGC and contained a ΦC31-based phage integrase that appears to have a more relaxed specificity of integration [25, 27]. The perfect ΦC31-phage integration site was also identified in the *S. rimosus* R7 genome [28] (NCBI GenBank Accession number EF670587). However, there is no guarantee that different *S. rimosus* strains have identical overall genome landscapes [29]. As this knowledge and the nucleotide alignment score of the *S. rimosus* R7 perfect *attB* site and the *S. rimosus* ATCC 10970 genome were not 100 %, this urged us to define the chromosomal location in the M4018 strain. Then by applying the plasmid rescue technique, we were able to identify the sequences in the vicinity of the the ΦC31 *attB* sites in the M4018 parental strain.

We also carried out the plasmid rescue to identify the correct integration sites. We identified two *attB* sites, one of which did not have a perfect *attB* sequence (pseudo *att*). Our results support the data of Combes et al. (2002), who demonstrated that perfect *attB* sites are very conserved throughout *Streptomyces*, while pseudo *attB* sites can show more variability as recognition sites [25] (Additional file 1: Figure S14). What is more, both the perfect and the pseudo *attB* sites appeared to be identical in the different *Streptomyces* spp. (Additional file 1: Figure S14). The perfect *attB* site lies within a putative gene that encodes a pirin-like protein, which has already been identified in ATCC 10970 [28] and in other *Streptomyces* spp. [25–28].

Interestingly, both of the integration sites are located in the core region of the *S. rimosus* chromosome, which is generally considered to be a ‘stable region’. This might thus be the reason for the relatively high strain stability that was observed. As we had two integration sites, with both located in the stable core region of the chromosome (Fig. 1), although still far apart (940 kb), ΦC31-integrase vectors were very useful tools for integration of multiple inserts into *S. rimosus*. In a few instances, in the single transformation experiments, we achieved simultaneous integration into both of these integration sites (e.g., the M4018 O51 transformant). Therefore, engineering of additional ΦC31-integrase sites in different regions of the chromosome combined with larger scale screening efforts might provide further increases in *otc* cluster copy numbers. Alternatively, other phage integrases with different *att* sites can be used, as reported in the literature [31–33].

Before introduction of the extra copies of the *otc* gene cluster into the selected strains, we deleted the *otc* BGC in ATCC 10970 and HP0508. For the M4018 lineage, we already had an M4018 progenitor strain available that lacked the *otc* BGC (15883S). We then re-introduced an entire *otc* BGC into the strains that lacked the *otc* BGC, which restored the initial production levels of OTC. We thus demonstrated that introduced *otc* BGC was fully functional and that all three of these strains with re-located *otc* BGC were morphologically and physiologically stable (Additional file 1: Figures S4, S6).

Surprisingly, there was a significant increase in OTC production in the ATCC 10970 strain where the *otc* gene cluster had been re-introduced, with some strains reaching > 1 g/L OTC production (Additional file 1: Figure S5). This represented a 5-fold titer compared to that of the ATCC 10970 parental strain. Clearly, the new integration site of the *otc* BGC had a beneficial effect on OTC production.

The native position of the *otc* BGC is ~ 600 kb away from one of the ends of the *S. rimosus* chromosome (Fig. 1), which is thought to be a reason for the pronounced instability of OTC production. Therefore, it has been argued that relocation of the *otc* gene cluster in the core region of *S. rimosus* chromosome might be beneficial for strain stability [29, 34]. Our data support this hypothesis. They also appear to be in agreement with the second part of the study, where a second copy of the *otc* BGC was introduced into the ATCC 10970 strain. Although two copies of the *otc* BGC were present in the ATCC 10970, these transformants did not perform better compared to the ATCC 10970ΔOTC with a re-located *otc* gene cluster (Fig. 2a, Additional file 1: Figure S5). Thus, two copies of the *otc* BGC did not appear to further contribute to the OTC titer increase. Alternatively, a lack of substrate supply might have an important effect on the increases in OTC titers.

In contrast, in the 15883S and HP0508 ΔOTC strains, we restored the OTC titer to similar levels to those observed in their parental strains, and thus no additional titer increase was seen. Importantly, we have demonstrated that the transformants are stable, which is an important validation of the engineering methodology and the tools used in this study.

As discussed earlier, an additional copy of the *otc* BGC in the native ATCC 10970 strain resulted in OTC titers > 1 g/L OTC, which represent a roughly 5-fold greater titer. On the other hand, the best performing M4018 transformants produced 2-3-fold higher OTC titers compared to the M4018 parental strain. Remarkably, under our testing conditions at a shake-flask scale, some M4018 transformants reached almost 80 % of the titer that can be achieved with the high OTC-producing HP0508 strain. On the other hand, some of the HP0508 transformants that carried an additional copy of the *otc* gene cluster showed an increase in their OTC titers of up to 30 % (Fig. 2c), which constitutes a significant achievement and demonstrates that this is a valuable methodology to apply to high OTC-producing strains.

We selected the highest titer transformants of the M4018 strain, and in an attempt to achieve the highest possible titer increases with the M4018 strain, we tested a larger number of independent M4018 transformants. Through PFGE electrophoresis and Southern blotting, we showed that these had an additional copy of the *otc* gene cluster. Indeed, the best producing strain, the O51 transformant, contained two additional copies of the *otc* gene cluster (Fig. 4), for a total of three copies of the *otc* gene cluster in this strain. As discussed earlier, this is possible as there are two phage ΦC31-integrase *att* sites in the chromosome of *S. rimosus*. We were then able to identify the exact location of the additional copies of the *otc* gene cluster through plasmid rescue (Fig. 4, Additional file 1: Figure S10).

Regardless of the strain, additional copies of the *otc* gene cluster clearly have a positive impact on OTC biosynthesis. A statistically significant increase was shown for the OTC titers when the selected transformants underwent re-testing. However, it was also important to evaluate the stabilities and reproducibilities of the transformants. The titer increase in the O51 transformant was the highest, and thus this transformant of the M4018 strain was used for the sub-culturing, to mimic the microbiological procedures that are used at the industrial scale. We followed the loss of the resistance marker on the integration plasmid (Additional file 1: Table S3), and observed relatively high stability for the integration and retention of the resistance marker in O51 transformant (Additional file 1: Figure S7).

To determine whether there had been any rearrangements of the *otc* gene cluster, the total DNA following its restriction underwent Southern blotting using rare cutting enzymes, followed by PFGE electrophoresis. A number of independent isolates from the O51 strain after the sub-culturing were tested, and generally they performed well. There was some variability in the OTC production when no thiostrepton was used at the vegetative stage, although the degree of variability was not much greater than the natural variability of the M4018 parental strain (Additional file 1: Figure S7). One independent segregant of the O51 transformant lost one copy of the *otc* gene cluster (Additional file 1: Figure S8). Interestingly, the titer of O51-4 is reduced on the medium without thiostrepton (Additional file 1: Figure S7b). Overall, these cultures were stable after numerous sub-culturing steps, despite having three copies of the *otc* gene cluster integrated into their chromosome.

Although there was ~ 28 kb of perfect homology among the integrated copies of the *otc* gene clusters, these were separated by 940 kb of DNA, as seen on PFGE (Additional file 1: Figure S10), which is probably what reduces the number of homologous recombination events that take place between them. Moreover, should a recombination event occur between two copies of the *otc* gene cluster, this would result in a large sequence deletion, which would very likely be lethal for the strain. Alternatively, and depending on the orientation of the *otc* gene clusters, a recombination event might result in inversion of the DNA fragment between two copies of the *otc* gene cluster; this would probably not change the architecture of the *otc* gene cluster. Perhaps, it is therefore not so unexpected to observe relatively high degree of stability.

Considering that an increase in a gene copy number should result in an increase in gene expression, we carried out RT-qPCR analysis of representative genes of the *otc* BGC. We carried out a comparative gene-expression study of the M4018 parental strain, the O51 transformant, and the HP0508 strain. Expression levels of the genes in the *otc* BGC in the M4018 strain were a lot lower than those in the HP0508 strain, as would be expected. Interestingly, the O51 transformant showed similar gene expression levels to the HP0508 high OTC-producer strain. Indeed, the expression levels of *otrA*, *otrB*, and *oxyS* were significantly higher in the O51 transformant compared to the HP0508 strain. It would appear that expression of the *otc* BGC in the O51 transformant is initiated earlier, and it is slightly higher at the beginning of the process, compared to the HP0508 strain (Fig. 5). However, gene expression of the *otc* cluster was sustained for longer in the HP0508 strain, potentially explaining its higher OTC titer at the end of the process. It is also worth noting (however difficult it might be to explain) that expression of the *oxyS* gene that encodes a carbon 6 and carbon 5 hydroxylase involved in the final stages of OTC biosynthesis was increased in the O51 transformant to a greater extent than for the other *otc* genes (Additional file 1: Figure S13). Amplification of the entire *otc* gene cluster appears to have resulted in overexpression of the regulatory and resistance genes in the cluster, which might also have additional effects on the expression of biosynthetic genes, as reported in the literature [35–37].

## Conclusions

It is important to explore and evaluate novel biosynthetic engineering approaches for further improvements to industrial high OTC-producing strains that can today reach very high titers of tens of g/L of the target product at the end of the process. Although advanced strain improvements and metabolic engineering approaches can be very productive, these approaches often do not result in additional titer increases when working with industrial high OTC-producing strains that have already undergone decades of intensive strain improvement regimes. The present study demonstrates that although increasing the copy number of a target BGC is technically very demanding, it represents a promising approach for the improvement of secondary metabolites in industrial settings. It is nonetheless important to clearly define a good strategy, to avoid DNA rearrangements and reduce general strain instability. As exemplified here, the proposed engineering work might result in varying degrees of success, which will probably depending on the strain background, substrate supply, and regulation and other properties of the host. In addition, the media, process conditions, and some properties of the target product will most likely have significant influences on the success of the selected strategy. Although this approach is technically very demanding and it requires a systematic effort, in combination with modern strain selection methods it represents very valuable methodology in industrial process development.

## Methods

### Strains and plasmids

The strains and plasmids used in this study are listed in Additional file 1:  Table S3. *E. coli* DH10β [38] was used for standard cloning procedures. *E. coli* ET12567/pUB307 [39] was used for conjugation between the *E. coli* and *S. rimosus* strains.

###  Media and culture conditions

*E. coli* strains were cultivated in 2× yeast extract–tryptone medium supplemented with 100 µg/mL ampicillin, 100 µg/mL apramycin, or 50 µg/mL kanamycin, as required, and cultivated at 28 °C. MS medium agar and TSB (1.7 % casein peptone, 0.3 % soy peptone, 0.25 % glucose, 0.5 % sodium chloride, 0.25 % dipotassium hydrogen phosphate; [40]) were used with incubations at 28 °C for sporulation and for cultivation of *S. rimosus* strains in liquid medium, respectively. For OTC production, *S. rimosus* was cultivated in GOTC-V seed medium (5 % tryptone, 1 % glucose, 0.1 % calcium carbonate, 0.5 % yeast extract), incubated for 24 h at 28 °C with 220 rpm shaking. Then, 10 % (v/v) of the seed culture was used for inoculation of the GOTC-P production medium (0.7 % MOPS, 4.2 % soy flour, 0.6 % ammonium sulfate, 0.2 magnesium chloride, 0.15 % sodium chloride, 0.73 % calcium carbonate, 2.8 % corn starch, 10 mL/L 1 % zinc sulfate solution, 3.75 mL/L 1 % manganese sulfate solution, pH 6.25). The strains were cultivated in production medium for 5 days at 28 °C with 220 rpm shaking, and 60 % humidity. Intergeneric conjugation was performed according to a previously reported procedure [39]. *S. rimosus* exconjugants were selected on MS medium plates containing nalidixic acid (25 µg/mL) and thiostrepton (30 µg/mL), or erythromycin (30 µg/mL). After selection, the colonies were patched onto MS medium containing the appropriate antibiotic and incubated for 7 days at 28 °C.

### High performance liquid chromatography

To quantify the OTC titers from the *S. rimosus* strains, 1 mL of fermentation broth was adjusted to pH 1.5-2.0 with 37 % HCl, and centrifuged at 13,000 rpm for 10 min. The supernatants were then subjected to HPLC analysis (UltiMate 3000 HPLC system) with a C-18 column (150 × 4.6 mm; 5 µm; 40 °C; Macherey-Nagel), with UV absorption detection at 270 nm, and a flow rate of 0.3 mL/min. The elution solvents were water with 0.1 % formic acid (solvent A) and acetonitrile (solvent B). The 30-min gradient elution was as follows: 0→20 min, 5 %→25 % B; 20→21 min, 25 %→90 % B; 21→25 min, 90 % B; 25→26 min, 90 %→5 % B; 26→30 min, 5 % B. The OTC titers were calculated as the mean titers from three parallel samples of each transformant and parental strain.

### Pulse‐field gel electrophoresis

The genomic DNA of the *S. rimosus* strains was prepared by inoculation of a plug from sporulating colonies on MS medium into 5 mL TSB medium. The cultures were grown at 28 °C with 220 rpm shaking for 24 h. Preparation of DNA samples from *S. rimosus* overnight cultures following the PFGE method [29]. The blocks containing gDNA were treated overnight with either restriction endonuclease *Ase*I or *Xho*I (1 block in 400 µL 1× fast digest buffer; 3 µL enzyme), and incubated overnight to avoid partial digestion. PFGE was performed in a Mapper apparatus (CHEF; Bio-Rad, USA) in 100 mL 1 % low melting point agarose gels run in 0.5× TBE buffer (10× TBE buffer: 108 g Tris base, 55 g boric acid, 40 mL 0,5 M EDTA, pH 8, dissolved in 1 L deionized H_2_O). The gels were stained with ethidium bromide (10 µL of 10 mg/mL ethidium bromide solution in 200 mL deionized H_2_O) for 20 min, and washed twice with 200 mL dH_2_O, and revealed (GEL Doc instrument).

### Probe design and southern blotting

Digoxigenin (DIG)-labeled DNA probes were generated with PCR DIG Probe Synthesis kits (Roche, Switzerland). The probes were labeled with DIG-dUTP (alkali-labile) by PCR, according to the Roche protocol. PFGE gels were transferred onto membranes and analyzed by Southern blotting with previously designed probes. The DNA was first transferred from the PFGE gels to a positively charged nylon membrane (Hybond Membrane; Amersham) through vacuum blotting. Briefly, each gel was incubated for 15 min in 0.25 M HCl (depurination), and then washed twice with deionized H_2_O, at 3 min each. The DNA was denatured with 0.5 M NaOH/ 1 M NH_4_Ac for 15 min, and transferred to the nylon membrane that was previously wet with 0.4 M NaOH. The DNA was transferred using a vacuum blotter (785; Bio-Rad, USA). After the transfer, the membrane was washed for 15 min in 1 M NH_4_Ac and dried for 20 min at 100 °C. These membranes were stored at room temperature until ready for hybridization. The DIG Nonradioactive System method (Roche, Switzerland) was used for nucleic acid labeling and detection.

### Cloning of the entire ***otc*** gene cluster from *S. rimosus* HP0508 by the single-step transformation associated recombination approach

The DNA fragments containing an entire *otc* gene clusters (Additional file 1: Figure S1) were cloned directly from the genomic DNA of the HP0508 strain using a single-step cloning approach based on TAR cloning in yeast [19]. The HP0508 strain was cultivated in TSB liquid medium. Genomic DNA was isolated following standard procedures [40], and was digested using *Spe*I as an overnight reaction at 37 °C. The digested DNA fragments were precipitated, washed with 70 % ethanol, and dissolved in 100 µL 20 % Tris-EDTA buffer. The primers OTC hook-up_F, OTC hook-up_R, OTC hook-down_F and OTC hook-down_R were use to amplify two homologous regions bordering the *otc* gene cluster by PCR (Phusion High-Fidelity DNA Polymerase; Thermo Scientific), both of which were ~ 1 kb in size. The two homologs were then digested by *Bam*HI and *Kpn*I and simultaneously cloned into the pYAC-ΦC31-Ts vector. Prior to TAR cloning, the pYAC-ΦC31-Ts vector with the two homologous ‘hooks’, was linearized with *Spe*I (Additional file 1: Figure S1). Preparation of the yeast spheroplast was following a previous protocol [41], and TAR cloning of the *otc* gene cluster from genomic DNA was performed following the protocol [19, 42].

The presence of an entire *otc* cluster cloned into the pYAC-ΦC31-Ts-OTC in the yeast transformants was confirmed initially by colony PCR screening using four strategic sequence-tagged site primer pairs specific for amplification of *otc* gene cluster sequences (STS-OT, STS-OS, STS-OH, STS-OP; Additional file1: Figure S1, Additional file 1: Table S1), which were located in the middle and extremities of the *otc* gene cluster. The transformants that showed positive PCR amplicons with all four sequence-tagged-site primer pairs were further analyzed by restriction analysis after re-transformation of the plasmid from the yeast transformants into *E. coli* DH10β. Plasmid DNA that presumably contained the entire *otc* gene cluster was isolated, and restriction analysis was completed from each individual *E. coli* transformant. Plasmid DNA was isolated from several independent *E. coli* transformants to confirm their genetic stabilities in both yeast and *E. coli*. All of the analyzed plasmid clones showed the same restriction enzyme pattern with *Sac*I restriction enzyme, corresponding to the expected restriction enzyme pattern of the cloned genomic fragment containing the entire *otc* gene cluster. Nonmethylated DNA of the pYAC-ΦC31-Ts-OTC plasmid was isolated from *E. coli* ET12567 and used for further transformation into different *S. rimosus* strains.

### Deletion of the entire *otc* gene cluster of *S. rimosus* ATCC 10970 and HP0508 strains

#### Deletion of the* otc* cluster in* S. rimosus* ATCC 10970

Deletion of the *otc* cluster from the genome of the ATCC 10970 strain was carried out using an iterative marker excision system (Additional file 1: Figure S15) [43]. For construction of the plasmid for deletion of the *otc* gene cluster, the BAC vector R3A4 from a BAC library (unpublished) was initially identified, which contained a 37 kb fragment with the entire *otc* gene cluster and the up and down region of the *otc* cluster. The antibiotic cassette (e.g., hygromycin, erythromycin) was flanked with the P-GG and B-CC sites for ΦC31 integrase, and amplified by PCR with primers HSU-OTC34 and HSU-OTC35 (Additional file 1: Table S1). Using the PCR-based λ-red recombination technique [44], the amplified *hyg*-*ery* cassette (Additional file1: Table S3) was used to delete the *otc* cluster on BAC R3A4 containing the entire *otc* gene cluster from the *otrB* gene to the *otrA* gene, on the other side of the *otc* gene cluster. The resulting BAC with the entire cluster substituted with the *hyg-ery* resistance cassette was introduced into ATCC 10970 by conjugation [39], and was selected for erythromycin resistance. Double crossover mutants were selected using blue-white [45]. Finally, the antibiotic cassette was excised from the chromosome of the selected double-crossover mutants by expression of the ΦC31 integrase from the pUWLint31 plasmid [43]. Cluster deletion was confirmed by PCR, using the HSU-OTC41 and HSU-OTC46 primers (Additional file 1: Table S1), followed by sequencing of the PCR-amplified fragment.

#### Deletion of the* otc* gene cluster in* S. rimosus* M4018

M4018 is a medium OTC-producer strain that was kindly provided by Prof. I.S. Hunter (University Strathclyde, Glasgow, UK). M4018 is a prototrophic strain that was used for commercial production of OTC by Pfizer [46]. The 15883S strain is an M4018 lineage strain derived by spontaneous deletion of the *otc* gene cluster during a strain improvement program [20].

#### Deletion of the* otc * gene cluster in* S. rimosus* HP0508

The strategy for deletion of the *otc* cluster in HP0508 (kindly provided by Prof. M. Guo, State Key Laboratory of Bioreactor Engineering, East China University of Science and Technology. Shanghai, China ) was based on homology recombination. The pAB13 plasmid was pKC1139 [39] derived plasmid with a thermo-sensitive replicon carrying 2.6 kb ‘up’ homology and 2.6 kb ‘down’ homology, which was used to delete the entire *otc* gene cluster, including the genes from o*xyA* and *oxyT*. Regulatory genes (*otcR, oxyTA1, otcG*) and genes responsible for resistance (*otrA*, *otrB*) were maintained in the genome. Homologies for the *otc* cluster deletion were amplified using the OTC_UP and OTC_DOWN primers, as listed in Additional file 1: Table S1. The up homology was cut with the *Xba*I and *Dra*I restriction enzymes, and the down homology was cut with the *Dra*I and *Eco*Ri restriction enzymes. Both of the homologs were simultaneously cloned into the pAB13 plasmid opened with *Xba*I and *Eco*RI. The nonmethylated plasmid was introduced into the HP0508 strain by transformation [40], and transformants were further selected on MS agar supplemented with erythromycin (30 µg/mL). The cultures were then subcultivated at 37 °C, five times without selection pressure (antibiotic) in TSB, and from the third day onward, plated for serial dilutions in MS plates. After 5 days of incubation, the colonies from the MS plates were patched onto MS plates without antibiotic and MS plates containing erythromycin. The primary recombinants were still resistant to erythromycin, while the secondary recombinants had lost their erythromycin resistance. The erythromycin-sensitive colonies were further confirmed by colony PCR amplification using the HSU-OTC41 and HSU-OTC46 primers (Additional file 1: Table S1).

### Reintroduction of the *otc* gene cluster in the *S. rimosus* ATCC 10970ΔOTC, 15883S, and HP0508 ΔOTC strains

The entire *otc* gene cluster located on the pYAC-ΦC31-Ts-OTC plasmid was transformed into *E. coli* ET12567/pUB307. In the next step, the plasmid was conjugated into ATCC 10970 **Δ**OTC, 15883S and HP0508 **Δ**OTC, via *E. coli* ET12567/pUB307–*S. rimosus* conjugation [39]. As a control, pYAC-ΦC31-Ts-h without the *otc* gene cluster was introduced into all three *otc*-deletion *S. rimosus* strains. Conjugants were patched on MS selective medium and cultured and analyzed as described in Sects. Media and culture conditions" and "High performance liquid chromatography".

### Introduction of additional *otc* gene cluster copies into the *S. rimosus* ATCC 10970, M4018, and HP0508 strains using ΦC31-phage integrase-mediated chromosomal integration

The pYAC-ΦC31-Ts-OTC plasmid (see Sect. "Cloning of the entire *otc* gene cluster from *S. rimosus* HP0508 by the single-step transformation associated recombination approach".) that contained the entire *otc* gene cluster was transformed into the ATCC 10970, M4018 or HP0508 strains via conjugation [39], as described in Sect. "Strains and plasmids". Exconjugants were selected, cultivated, and analyzed as described in Sects. "Media and culture conditions" and "High performance liquid chromatography". Sixteen morphologically stable M4018 transformants were selected for two re-test fermentations, to evaluate the consistency of the OTC production. In the first re-test fermentation method as described in Sect. "Media and culture conditions" was used. In the second re-test fermentation a plug from a sporulating colony on MS medium was inoculated into 5 mL GOTC-V medium and cultivated at 28 °C with 220 rpm shaking, for 24 h. After 24 h, 25 mL production medium (in a 250 mL glass flask) was inoculated with 10 % inoculum from seed cultures and cultivated at 28 °C with 220 rpm shaking and 60 % humidity for 5 days. The broth was then acidified and the OTC extracted as described in Sect. "High performance liquid chromatography". This test was repeated twice. Out of 16 M4018::otc transformants, six were selected for further analysis of genomic stability of the integrated plasmid and identification of the *otc* copies inserted into the *S. rimosus* genome.

### Identification of additional *otc* copies and ΦC31 attachment sites in *S. rimosus* M4018

Considering that the introduction of an additional copy of the *otc* cluster had such a positive effect on OTC production in medium OTC-producing M4018 strain, six independent M4018transformants (O4, O24, O45, O51, O64, O83, O97) that showed the highest increases in the OTC titers were analyzed by PFGE and Southern blotting to evaluate the number of *otc* copies integrated into the genome of the M4018 strain. The DIG-labelled probes were prepared according to the manufacturer instructions (Roche). The analysis was conducted as described in Sects. "Pulse-field gel electrophoresis" and "Probe design and Southern blotting". As a control, the M4018 strain was also transformed with empty pYAC-ΦC31-Ts-h plasmid.

### Evaluation of genetic stability of the *S. rimosus* M4018 strain containing multiple copies of the ***otc*** gene cluster

The genetic stability of M4018::otc transformant O51 obtained that showed significant increases in OTC titer and containing multiple copies of the chromosomally integrated *otc* gene cluster was tested with two independent procedures: OTC titer measurements and PFGE analysis.

The genetic stability of M4018::otc was tested by monitoring the OTC titers in the M4018 strain, the M4018::otc O51 transformant, and seven colonies that were derived from colony O51 and M4018::YAC-h transformants. Seven independent and morphologically stable O51-derived colonies were obtained by streaking colony O51 on MS medium to single colonies. Seven single colonies were afterwards patched on MS media to obtain more biomass. A plug of spores from patches on MS plates were inoculated in 5 mL TSB medium without and with thiostrepton, and were cultivated at 28 °C with 220 rpm shaking. After 24 h, 10 % of the culture was used to inoculate 5 mL GOTC production medium, which was then cultivated at 28 °C with 220 rpm shaking and at 60 % humidity, for 5 days. After 5 days the OTC was extracted from the production broth and analyzed as described in Sect. "High performance liquid chromatography".

Finally here, the genetic stability of the selected M4018::otc strains was tested by analysis of the genome via PFGE. Seven colonies derived from the O51 transformant, as described above, were inoculated with a plug from sporulating colonies on MS medium in 5 mL TSB medium, and cultivated at 28 °C with 220 rpm shaking. After 24 h cultivation, the transformants were analyzed on PFGE after restriction with *Xho*I or *Ase*I rare cutter enzymes, as described in Sect. "Pulse-field gel electrophoresis". The M4018::otc O51 transformant was taken as the control.

### Location of ΦC31 attachment sites containing the integrated *otc* gene cluster in the *S. rimosus* M4018 strain

To identify the ΦC31 *attB* sites present in the *S. rimosus* chromosome, where ΦC31 integrase derived plasmids awere ctually integrated, the pYAC-ΦC31-Ts plasmid (without the *otc* gene cluster) containing the ΦC31 integrase and the *attP* site was transformed into M4018 via conjugation, as described in Sect. "Media and culture conditions". Genomic DNA of the M4018::YAC transformants, as well as the control M4018, M4018 15883S strains and the ATCC 10970 strains, was prepared for PFGE analysis as described in Sect. "Pulse-field gel electrophoresis". The genomic DNA was treated with the *Ase*I restriction enzyme. To determine the ΦC31 *attB* sites in *S. rimosus*, the pYAC-ΦC31-Ts plasmid with the additional flanking sequences around integration site was rescued from the M4018 chromosome of selected transformants to identify the DNA sequence targeted by the ΦC31 recombinase. The M4018::YAC transformants and *the* M4018 strains had their genomic DNA isolated following standard procedures [40]. Here, 1–2 µg gDNA of all strains was digested independently overnight with 2 µL *Sac*I in a 20 µL reaction. The plasmid rescue protocol was performed following previous instructions [22]. Ligation of digested DNA was carried out overnight at 16 °C after addition of 2 µL 10X ligase buffer, 1 µL T4 DNA ligase, and completed with deionized H_2_O to 20 µL. Then 2 µL of the ligation mix were transformed into *E. coli* DH10β, and the colonies were selected against ampicillin. Plasmid DNA was isolated from the colonies obtained, and its restriction pattern was analyzed with *Nco*I/*Bam*HI. The plasmids that displayed different restriction patterns were sequenced with the primer ΦC31 fish Rv (Table S1), to analyze the integration of the *attB* sites present in the *S. rimosus* chromosome. DIG-labeled DNA probes for Southern blotting were generated from the rescued plasmids to identify the location of the *attB* sites specific for ΦC31 recombinase in the M4018 chromosome. Primers Fw P1, Rv P1, Fw P2 and Rv P2 (Additional file 1: Table S1) were designed according to the DNA sequences obtained from the rescued plasmids, and the rescued plasmids were also used as PCR templates to generate the hybridization probes. The Southern blotting analysis followed the protocol described in Sect. "Probe design and Southern blotting".

### Expression studies of selected ***oxy*** genes by RT-qPCR

The M4018 and HP0508 strains were cultured in GOTC production medium according to the procedure described in Sect. "Media and culture conditions". The cells were sampled from cultured broth at 12, 20 or 24 (HP and M4108 strains, respectively), 36, 48, 72 and 96 h. The samples were fixed by addition of 5-fold the amount of the fixative (ethanol:phenol 96:4), and stored at − 80 °C. Total RNA and cDNA were prepared as described previously [47].

Genes involved in regulation (*otrR* and *oxyTA1*), resistance (o*trB*), minimal PKS gene (*oxyA*), and post-PKS gene (*oxyS*) were chosen as the targets, and the primers and the TaqMan MGB probes (Additional file 1: Table S1) were designed as Custom TaqMan Gene Expression assays (Thermo Fisher Scientific). The RT-qPCR was set-up as described previously [47]. The standard curve method was used for quantification of relative gene expression (http://quantgenius.nib.si; 48), with 16 s rRNA [47] as a reference gene.

### Statistical analysis

When the M4018 transformants were compared with each other, statistical analysis was performed using analysis of variance (ANOVA), with SPSS windows version 26.0 (SPSS Inc., Chicago, IL, USA). Mann-Whitney U-tests were used for the comparisons of the OTC titers from the parental strain and their transformants, at the level of P < 0.05 [21].

## Supplementary Information

**Additional file 1: Table S1:** Primers used in this study, and their respective sequences. RT-qPCR TaqMan MGB probes (P) were labeled with FAM at 5’, and nonfluorescent quencher (NFQ) at 3’. **Table S2:** Relative gene expression data for a reference (16S rRNA) and 5 target genes. Relative copy numbers (obtained by quantification using standard curve), relative copy numbers, normalized to the reference gene and relative normalized copy numbers, scaled to the lowest expression value for each individual gene (presented in Figure 5), are shown. Relative copy numbers are not comparable between genes. **Table S3:** Strains and plasmids used in this study. **Figure S1.** Schematic presentation of the plasmid containing the entire otc gene cluster, obtained via a single-step transformation-associated recombination cloning approach. The homologies used to clone the entire gene cluster are located externally at both ends of the otc gene cluster, and are labeled in blue. The *S. cerevisiae*/ *E. coli*/ *Streptomyces* shuttle vector is approximately 7400 bp long, each homology for homologous recombination is approximately 1 kb long, and otc cluster is approximately 28 kb long. The black arrows indicate primers used to screen for the correct plasmid. The red arrows above left and right homology, Hook-Up and Hook-Do, respectively, indicate primers used to create Southern blotting probes, which were used for otc cluster identification in Figure 4. **Figure S2:** SacI restriction analysis of individual plasmid constructs that were assumed to contain the entire otc gene cluster. The restriction pattern of pYAC-ΦC31-Ts-OTC (38408 bp) digested with SacI, demonstrated seven bands of 11638 bpand 10515 bp (these two appear as one band on the agarose gel), 5029 bp, 4373 bp, 4298 bp, 1885 bp, and 670 bp clones. (a-e) Transformants obtained from genomic DNA of the individual yeast transformants previously selected by colony PCR. (a1-a4) Individual plasmid rescue clones from *E. coli*. **Figure S3:**Morphological properties of the wild-type *S. rimosus* ATCC 10970 strain, the M4018 medium OTC-producing strain, and the HP0508 high OTC-producing strain, and of the same strains with an entire otc cluster deleted. (A, B) Upper of the MS plates, showing *S. rimosus* ATCC 10970, M4018, and HP0508 (A), and showing the *S. rimosus* ΔOTC mutants (B). (C, D) Bottom of MS plates, showing of *S. rimosus* ATCC 10970, M4018 and HP0508 (C), and showing the *S. rimosus* ΔOTC mutants (D). All three of the strains show slightly darker brown mycelia, as observed at the bottom of the plate. The colour has been correlated with OTC production; however, the pigment is of an unknown source, and its intensity does not directly correspond to the OTC titer, as the M4018 strain, which is a medium OTC-producer, shows the most intense dark color. The dark pigmentation disappears following otc gene cluster deletion. All three of the strains produce white spores on top of the mycelia; however, sporulation is reduced with higher OTC production, and HP0508 shows the lowest number of spores, indicating non-homogenous growth of the spores (Figure S3A, B). **Figure S4:** HPLC analysis of the OTC produced by different *S. rimosus* strains and their respective OTC deletion mutants. (A) ATCC 10970. (B) ATCC 10970ΔOTC. (C) M4018. (D) 15883S. (E) HP0508. (F) HP0508ΔOTC. The arrows indicate OTC with a retention time of approximately 18.2 min. OTC was quantified using a standard and calculated from the area under curve. **Figure S5:** OTC titer following complementation of the *S. rimosus* ATCC10970 ΔOTC, 15883S, and HP0508 ΔOTC deletion mutants with an entire otc gene cluster. Blue bars, relative OTC titers of the parent *S. rimosus* strains: ATCC 10970, M4018, HP0508. Orange bars, relative OTC titers of the complemented ΔOTC mutants. **Figure S6:** OTC titers from the independent transformants carrying the pYAC-ΦC31-Ts-h control plasmid (i.e., without the otc cluster) in *S. rimosus* ATCC 10970, M4018, and HP0508. Blue bars, relative OTC titers of the parent *S. rimosu*s strains: ATCC 10970, M4018, HP0508. Orange bars, relative OTC titers of the independent pYAC-ΦC31-Ts-h transformants. The significance of the variability was tested with two-tailed Mann-Whitney tests. No significant variability was detected for the ATCC 10970 strain transformed with the control plasmid (P > 0.05), while significant variability was detected for the M4018 and HP0508 transformants with the control (P < 0.05). **Figure S7:** OTC titers of the initial O51 transformant and of seven colonies derived from the O51 colony. M4018 parental strain and pYAC-ΦC31-Ts-h O4 were taken as the control strains. (A) Vegetative medium with thiostrepton. (B) Vegetative medium without thiostrepton. The titers were measured after 5 days of fermentation in 5 mL GOTC production medium. * P < 0.05 (vs. control O4 parental strain; two-tailed Mann-Whitney test). **Figure S8:** The stabilities of the integrated copies of the otc gene clusters in the initial O51 transformant and for its derived single colonies (O51-1-7) after vegetative growth in TSB medium without thiostrepton (A) XhoI digestion. (B) AseI digestion. L, Lambda concatamers, M, *S. cerevisiae* chromosomes. Right: Southern blotting of the PFGE gels, hybridized with probes generated from external homologs of pYAC ΦC31-Ts-h. Arrows indicate the otc cluster that is present in the band. Asterisk indicates colony O51-4, which lost one copy of the otc cluser during the growth in TSB medium. **Figure S9:** Schematic representation of plasmid pYAC-ΦC31-Ts and its integration into the *S. rimosus* chromosome. **Figure S10:** (A) Pulse-field gel electrophoresis (PFGE) analysis of chromosomal DNA digested with AseI. (B) Membrane treated with a probe generated with the Fw P1 and Rv P1 primers (Table S2). (C) Membrane treated with a probe generated with the Fw P2 and Rv P2 primers. Lanes: M, Saccharomyces cerevisiae chromosome marker; 1, ATCC 10970; 2, 15883S; 3, M4018; 4. 5. 6. 7,* S. rimosus*: YAC transformants. L: Lambda PFGE ladder. **Figure S11:** Possible integration events of plasmid pYAC-ΦC31-Ts. (A) The largest fragment of the *S rimosus* M4018 genome (2.3 Mb), after restriction with an AseI restriction nuclease. This carries both perfect and pseudo ΦC31 attB sites. (B) Single integration event at the perfect ΦC31 attB. In Figure S10, this corresponds to transformants in lane 6 and a subpopulation of transformants in lanes 4 and 5. (C) Double integration event. In Figure S10, this corresponds to transformants in lane 7 and a subpopulation of transformants in lanes 4 and 5. Plasmid pYAC-ΦC31-Ts contains two AseI restriction sites; however, as they are close together (approximately 1 kb), they cannot be separated by PFGE, and are thus presented as one AseI site. **Figure S12:** (A) Restriction patterns of the pYAC-ΦC31-Ts plasmid and rescued plasmids generated with BamHI/NcoI. pYAC-ΦC31-Ts vector (7.5 kb) digested with BamHI/NcoI shows titers with two bands of 1.7 kb and 5.8 kb. L1, Lambda DNA/HindIII ladder; L2, 1 kb gene ruler; C, pYAC-ΦC31-Ts BamHI/NcoI; 1-14, rescued plasmids digested with BamHI/NcoI. All of the plasmids show the corresponding 5.8 kb band (core structure of pYAC-ΦC31-Ts); the rescued plasmids showed two different patterns compared to the base line in pYAC-ΦC31-Ts vector. (B) Sequences of the attachment sites recognized by ΦC31 int/attP recombinase in the *S. rimosus* genome. Comparison of attP, attB, and pseudo attB sites before integration and modification of attB (attL and attR) sites after integration. The attP sequence is underlined, conserved nucleotides of the attB sites are highlighted, and the core region 5´TT where cross-over occurs is indicated inside the black box. **Figure S13:** Ratios of the selected gene expression in the *S. rimosus* O51 transformant, compared to the M4018 parent strain. **Figure S14:** (A) Alignment of DNA sequences corresponding to the perfect ΦC31 attB sites identified in the different *Streptomyces* strains: *Streptomyces* cinnamonensis (cinnamonensis); *Streptomyces* griseus (griseus); Kitasatospora aureofaciens (aureofaciens);* Streptomyces rimosus* ATCC 10970 (ATCC10970); *Streptomyces rimosus* M4018 (M4018); *Streptomyces rimosus* R7 (r7); *Streptomyces* hygroscopicus (hygroscopicus); *Streptomyces* clavuligerus (clavuligerus); *Streptomyces* coelicolor (coelicolor). (B) Alignment of the DNA sequences of the pseudo ΦC31 attB sites identified in the different *Streptomyces rimosus* and *Streptomyces* coelicolor strains. The core TT signature where the crossover occurs is highlighted inside the gray box. Nucleotides sharing identity are denoted with asterisks. **Figure S15:** Schematic presentation of the otc cluster deletion in *S. rimosus* ATCC 10970 using an iterative marker excision system.

## Data Availability

All data generated or analyzed during this study are included in this published article and its supplementary information files.
