# Supplementary material for: Multiple copies of the oxytetracycline gene cluster in selected Streptomyces rimosus strains can provide significantly increased titers
Source: Microb Cell Fact. 2021 Feb 17;20:47. doi: 10.1186/s12934-021-01522-5 (PMC7890619; doi:10.1186/s12934-021-01522-5)
Supplement: Supplementary file 1 — Additional file 1: Table S1: Primers used in this study, and their respective sequences. RT-qPCR TaqMan MGB probes (P) were labeled with FAM at 5’, and nonfluorescent quencher (NFQ) at 3’. Table S2: Relative gene expression data for a reference (16S rRNA) and 5 target genes. Relative copy numbers (obtained by quantification using standard curve), relative copy numbers, normalized to the reference gene and relative normalized copy numbers, scaled to the lowest expression value for each individual gene (presented in Figure 5), are shown. Relative copy numbers are not comparable between genes. Table S3: Strains and plasmids used in this study. Figure S1. Schematic presentation of the plasmid containing the entire otc gene cluster, obtained via a single-step transformation-associated recombination cloning approach. The homologies used to clone the entire gene cluster are located externally at both ends of the otc gene cluster, and are labeled in blue. The S. cerevisiae/ E. coli/ Streptomyces shuttle vector is approximately 7400 bp long, each homology for homologous recombination is approximately 1 kb long, and otc cluster is approximately 28 kb long. The black arrows indicate primers used to screen for the correct plasmid. The red arrows above left and right homology, Hook-Up and Hook-Do, respectively, indicate primers used to create Southern blotting probes, which were used for otc cluster identification in Figure 4. Figure S2: SacI restriction analysis of individual plasmid constructs that were assumed to contain the entire otc gene cluster. The restriction pattern of pYAC-ΦC31-Ts-OTC (38408 bp) digested with SacI, demonstrated seven bands of 11638 bpand 10515 bp (these two appear as one band on the agarose gel), 5029 bp, 4373 bp, 4298 bp, 1885 bp, and 670 bp clones. (a-e) Transformants obtained from genomic DNA of the individual yeast transformants previously selected by colony PCR. (a1-a4) Individual plasmid rescue clones from E. coli. Figure S3:Morphological proper [file 12934_2021_1522_MOESM1_ESM.doc]

**Supporting information for:**

**Multiple copies of the oxytetracycline gene cluster in selected** ***Streptomyces rimosus* strains can provide significantly increased titers**

Špela Pikl^1^, Andrés Felipe Carrillo Rincón^1^, Lucija Slemc^1^, Dušan Goranovič^2^, Martina Avbelj^1^, Krešimir Gjuračić^2^, Hilda Sucipto^3,4^, Katja Stare^5^, Špela Baebler^5^, Martin Šala^6^, Meijin Guo^7^, Andriy Luzhetskyy^3,4^, Hrvoje Petković^1*^, Vasilka Magdevska^2*^

^1^ Department of Food Science and Technology, Biotechnical Faculty, University of Ljubljana, Ljubljana, Slovenia

^2^ Acies Bio, d.o.o., Tehnološki Park, Ljubljana, Slovenia

^3^ Pharmazeutische Biotechnologie, Universität des Saarlandes, Saarbrücken, Germany

^4^ Helmholtz-Institut für Pharmazeutische Forschung Saarland, Saarbrücken, Germany

^5^ National Institute of Biology, Večna pot 111, Ljubljana, Slovenija

^6^ National Institute of Chemistry, Hajdrihova 19, SI-1000 Ljubljana, Slovenia

^7^ State Key Laboratory of Bioreactor Engineering, East China University of Science and Technology. Shanghai, China

* To whom correspondence should be addressed: Hrvoje Petković, Biotechnical faculty, University of Ljubljana, Jamnikarjeva 101, 1000, Ljubljana, Slovenia; tel: +38640488498; E-mail: [hrvoje.petkovic@bf.uni-lj.si](mailto:hrvoje.petkovic@bf.uni-lj.si); Vasilka Magdevska, Acies Bio d.o.o., Tehnološki park 21, 1000 Ljubljana, Slovenia, +38659075995, E-mail: [vasilka.magdevska@aciesbio.com](mailto:vasilka.magdevska@aciesbio.com)

**Table S1:** Primers used in this study, and their respective sequences. RT-qPCR TaqMan MGB probes (P) were labeled with FAM at 5’, and nonfluorescent quencher (NFQ) at 3’.

| **Primer name** | | **Sequence (5´ to 3´)** | | | | **Description** | | | |
| --- | --- | --- | --- | --- | --- | --- | --- | --- | --- |
| OTC_UP_Fw_XbaI | | AAAATCTAGAGTGGCAGAACACCCCGGCAC | | | | Primer pairs specific for deletion of OTC cluster in HP0508 | | | |
| OTC_UP_Rv_DraI | | AAAAATTTAAACGTCGCGCGTCATGGATCT | | | |
| OTC_Down-Fw_DraI | AAAAATTTAAACGTCCAGCAGCGTCGAGGAG | | | |  | | | | |
| OTC_Down_Rv_EcoRI | AAAAAGAATTCCATACCTCCACGGCGCGTC | | |  | | | | |  |
| OTC hook-up_F | | ATATAGGATCCCCCCCGTACACACACGCTT | | | | Primer pairs for TAR cloning OTC homology amplification | | | |
| OTC hook-up_R | | ATATAACTAGTACTGCATGGTGCGGTCCCAGGCGCG | | | |
| OTC hook-down_F | ATATAACTAGTCATCCTGCACGTGCTCAAGCGCGTG | | | |  | | | | |
| OTC hook-down_R | ATATAGGTACCAAGCGTGCGTGGGGGTGCCTCTCCC | | |  | | | | |  |
| STS-OT | | TGTCCAAGATCCATGACGCGCGA | | | | STS primer pairs specific for amplification of *OxyA* | | | |
|  | | | TCATCGATTTGATGGAGCTGATCGG | | | |  |  | |
| STS-OS | | TATGCGGTACGACGTGGTGATCG | | | | STS primer pairs specific for amplification of *OxyS* | | | |
|  | | | TCACATGGCGGGCGGCCCGAA | | | |  |  | |
| STS-OH | | TTGTCGCACTTGAAGACGTCCTTGAC | | | | STS primer pairs specific for amplification of *OxyH* | | | |
|  | | | CGCATCGACGACGTGCTGTCGC | | | |  |  | |
| STS-OP | | TCAGGGAATCCGGTACCCCTCACCC | | | | STS primer pairs specific for amplification of *OxyP* | | | |
|  | | | GCCTTCGTGTCCGCCGTCAT | | | |  |  | |
| HSU-OTC34 | | CGGGCGGAAATCCGCCGCGGGGCGCGGTCCGGTGCCGGTTGTAGGCTGGAGCTGCTTCG | | | | For Red/ET mediated gene deletion within R3A4 construct. Underlined sequences anneals to hyg-ery IMESE cassette. | | | |
| HSU-OTC35 | | CATGACGCGCGACGCGTCGTAATCACAGGGATCGGCGTGTTCCGGGGATCCGTCGACCC | | | |
| HSU-OTC41 | | GCCATCTCCTCGATCGTC | | | | For PCR confirmation of OTC cluster deletion | | | |
| HSU-OTC46 | | GGAGTATCTGGCCGAGGAC | | | |  | | | |
| φC31 fish Rv | | CGTTGGCGCTACGCTGTGT | | | | For analysis of the integration *attB* sites present in S. rimosus chromosome. | | | |
| Fw P1 | | CACCGCGGCTTCGAGACC | | | | Primers used to create Southern blot probes to identify *attB* location | | | |
| Rv P1 | | CTCCGGCCTCAGTGGCCGT | | | |
| Fw P2 | CACGACGAAGGCGCTGATGC | | | |  | | | | |
| Rv P2 | TCACGTATCCCGCGTGTCCTG | | |  | | | | |  |
| OXYS_F | | GCGGGAGCTGATGATCGA | | | | Primers and probe for analysis of *OxyS* with RT-qPCR | | | |
| OXYS_R | | GAGGCCCGCCAGATAGC | | | |
| OXYS_P | FAM_ACGTCCCGGAGACCAA_NFQ | | | |  | | | | |
| OXYA_F | | CGCCATCTCCCCCATCAC | | | | Primers and probe for analysis of *OxyA* with RT-qPCR | | | |
| OXYA_R | | GTCTCCGGCTCGTCGTT | | | |
| OXYA_P | FAM_AAGGCCACCACGCCGC_NFQ | | | |  | | | | |
| OXYTA1_F | | GTCCAGCAGACCGTCGAG | | | | Primers and probe for analysis of *OxyTA1* with RT-qPCR | | | |
| OXYTA1_R | | GGTCCTGCGGGTTGGG | | | |
| OXYTA1_P | FAM_CCCTCGGCCTTCAGC_NFQ | | | |  | | | | |
| OTCR_F | | AGTACCCCTACGACGAATGGT | | | | Primers and probe for analysis of *OtcR* with RT-qPCR | | | |
| OTCR_R | | GCGGCCGGCTTTGC | | | |
| OTCR_P | FAM_TCCACGCCCAGCTCAT_NFQ | | | |  | | | | |
| OTRB_F | | GGGCTGGCGGTCTTCAT | | | | Primers and probe for analysis of *OtrB* with RT-qPCR | | | |
| OTRB_R | | CCCCAGCGGCAGGAT | | | |
| OTRB_P | FAM_CCCCGTCGAGCTGC_NFQ | | | |  | | | | |

**Table S2:** Relative gene expression data for a reference (16S rRNA) and 5 target genes. Relative copy numbers (obtained by quantification using standard curve), relative copy numbers, normalized to the reference gene and relative normalized copy numbers, scaled to the lowest expression value for each individual gene (presented in Figure 5), are shown. Relative copy numbers are not comparable between genes.

| **Sample** | | **Relative copy numbers** | | | | | | **Normalized relative copy numbers** | | | | | **Scaled normalized relative copy numbers** | | | | |
| --- | --- | --- | --- | --- | --- | --- | --- | --- | --- | --- | --- | --- | --- | --- | --- | --- | --- |
| **strain** | **time** | **16S rRNA** | **otcR** | **otrB** | **oxyTA1** | **oxyA** | **oxyS** | **otcR** | **otrB** | **oxyTA1** | **oxyA** | **oxyS** | **otcR** | **otrB** | **oxyTA1** | **oxyA** | **oxyS** |
| **HP0508** | **12h** | 910.8 | 273.3 | 85.4 | 227.8 | 134.1 | 123672 | 0.300 | 0.094 | 0.250 | 0.147 | 135.8 | 113.0 | 32.4 | 69.0 | 28.4 | 31.0 |
|  | **20h** | 820.4 | 94.7 | 135.8 | 159.0 | 194.4 | 142799 | 0.115 | 0.166 | 0.194 | 0.237 | 174.1 | 43.5 | 57.2 | 53.5 | 45.7 | 39.7 |
|  | **36h** | 621.1 | 57.6 | 155.5 | 191.7 | 132.4 | 57831 | 0.093 | 0.250 | 0.309 | 0.213 | 93.1 | 34.9 | 86.4 | 85.1 | 41.1 | 21.2 |
|  | **48h** | 987.7 | 40.5 | 125.9 | 121.0 | 56.1 | 29330 | 0.041 | 0.127 | 0.123 | 0.057 | 29.7 | 15.4 | 44.0 | 33.8 | 10.9 | 6.8 |
|  | **72h** | 712.9 | 60.8 | 95.8 | 103.3 | 54.6 | 25773 | 0.085 | 0.134 | 0.145 | 0.077 | 36.2 | 32.1 | 46.4 | 40.0 | 14.8 | 8.2 |
|  | **96h** | 986.2 | 75.9 | 53.8 | 40.5 | 103.9 | 57839 | 0.077 | 0.055 | 0.041 | 0.105 | 58.6 | 29.0 | 18.8 | 11.3 | 20.3 | 13.4 |
| **M4018** | **12h** | 1084.7 | 28.6 | 55.6 | 32.7 | 197.5 | 94216 | 0.026 | 0.051 | 0.030 | 0.182 | 86.9 | 9.9 | 17.7 | 8.3 | 35.1 | 19.8 |
|  | **24h** | 737.7 | 23.5 | 46.6 | 28.0 | 176.5 | 58831 | 0.032 | 0.063 | 0.038 | 0.239 | 79.8 | 12.0 | 21.8 | 10.5 | 46.1 | 18.2 |
|  | **36h** | 1392.4 | 15.7 | 15.4 | 12.3 | 52.6 | 36345 | 0.011 | 0.011 | 0.009 | 0.038 | 26.1 | 4.2 | 3.8 | 2.4 | 7.3 | 6.0 |
|  | **48h** | 1457.4 | 12.0 | 9.2 | 8.9 | 36.0 | 32016 | 0.008 | 0.006 | 0.006 | 0.025 | 22.0 | 3.1 | 2.2 | 1.7 | 4.8 | 5.0 |
|  | **72h** | 1641.3 | 5.1 | 6.3 | 6.0 | 11.9 | 8751 | 0.003 | 0.004 | 0.004 | 0.007 | 5.3 | 1.2 | 1.3 | 1.0 | 1.4 | 1.2 |
|  | **84h** | 1530.6 | 4.1 | 4.4 | 7.0 | 7.9 | 6713 | 0.003 | 0.003 | 0.005 | 0.005 | 4.4 | 1.0 | 1.0 | 1.3 | 1.0 | 1.0 |
| **M4018-O51** | **12h** | 359.1 | 303.2 | 423.6 | 254.5 | 962.6 | 668672 | 0.844 | 1.179 | 0.709 | 2.680 | 1862.0 | 317.8 | 407.3 | 195.5 | 516.5 | 424.6 |
|  | **24h** | 625.9 | 126.2 | 286.3 | 106.3 | 686.3 | 763697 | 0.202 | 0.457 | 0.170 | 1.096 | 1220.1 | 75.9 | 158.0 | 46.8 | 211.3 | 278.2 |
|  | **36h** | 1186.6 | 124.7 | 109.0 | 50.4 | 346.1 | 890631 | 0.105 | 0.092 | 0.042 | 0.292 | 750.6 | 39.6 | 31.7 | 11.7 | 56.2 | 171.1 |
|  | **48h** | 1100.2 | 119.9 | 73.7 | 28.4 | 282.7 | 995404 | 0.109 | 0.067 | 0.026 | 0.257 | 904.7 | 41.0 | 23.1 | 7.1 | 49.5 | 206.3 |
|  | **72h** | 1405.6 | 34.6 | 10.2 | 6.4 | 50.6 | 330426 | 0.025 | 0.007 | 0.005 | 0.036 | 235.1 | 9.3 | 2.5 | 1.2 | 6.9 | 53.6 |
|  | **84h** | 1341.7 | 26.8 | 8.0 | 6.9 | 23.4 | 163498 | 0.020 | 0.006 | 0.005 | 0.017 | 121.9 | 7.5 | 2.1 | 1.4 | 3.4 | 27.8 |

**Table S3:** Strains and plasmids used in this study.

| **Strain or plasmid** | **Characteristics** | **Reference or source** |
| --- | --- | --- |
| *S. rimosus* |  |  |
| ATCC 10970 | *S. rimosus* ATCC10970 OTC producing strain (WT strain) | (1) |
| ATCC 10970ΔOTC | *S. rimosus* ATCC10970 WT strain with deleted *otc* cluster | This work |
| ATCC 10970ΔOTC::otc | *S. rimosus* ATCC10970 WT strain with deleted *otc* cluster and complemented with *otc* cluster | This work |
| ATCC 10970::otc | *S. rimosus* ATCC10970 WT strain with additional *otc* cluster | This work |
| ATCC 10970::YAC-h | *S. rimosus* ATCC10970 WT strain with control plasmid pYAC- ΦC31-Ts-h | This work |
| M4018 | *S. rimosus* M4018 OTC medium producing strain | (2)  DSM 105900 |
| S15883S (also designated as M4018ΔOTC) | *S. rimosus* M4018 strain lineage with spontaneous deletion of OTC cluster | (3) |
| S15883S::otc | *S. rimosus* M4018 strain lineage with spontaneous deletion of OTC cluster complemented with *otc* cluster | This work |
| M4018::otc | *S. rimosus* M4018 OTC medium producing strain with additional *otc* cluster | This work |
| M4018::YAC-h | *S. rimosus* M4018 OTC medium producing strain with control plasmid pYAC- ΦC31-Ts-h | This work |
| M4018::YAC | *S. rimosus* M4018 OTC medium producing strain with control plasmid pYAC- ΦC31-Ts | This work |
| HP0508 | *S. rimosus* HP0508 OTC high producing strain | This work |
| HP0508ΔOTC | *S. rimosus* HP0508 strain with deleted *otc* cluster | This work |
| HP0508ΔOTC::otc | *S. rimosus* HP0508 strain with deleted OTC cluster complemented with otc cluster | This work |
| HP0508::otc | *S. rimosus* HP0508 OTC high producing strain with additional *otc* cluster | This work |
| HP0508::YAC-h | *S. rimosus* HP0508 OTC high producing strain with control plasmid pYAC- ΦC31-Ts-h | This work |
| *E.coli* |  |  |
| DH10β | General cloning host | (4) |
| ET 12567/pUB307 | Strain for intergenic conjugation | (5) |
| ET 12567 | Cloning host | (6) |
| *S. cerevisiae* |  |  |
| INV*Sc*1 | Strain used for TAR cloning  MATα, his3-d1, Leu2, trp1–289, ura3–52 | Invitrogen, Inc. |
| Plasmids |  |  |
| pYAC-ΦC31-Ts | *Bla* gene, pMB1 *ori*, Am^R^, trp1/ARS1, CEN4, Ts^R^; ΦC31, | This work |
| pYAC-ΦC31-Ts-OTC | *Bla* gene, pMB1 *ori*, Am^R^, trp1/ARS1, CEN4, Ts^R^; ΦC31, contains whole OTC | This work |
| pYAC-ΦC31-Ts- h | *Bla* gene, pMB1 *ori*, Am^R^, trp1/ARS1, CEN4, Ts^R^; ΦC31, containing homology »hooks« | This work |
| pAB13 | pKC1139 derived plasmid, Ap^R^, Erm^R^; contains thermo sensitive replicon | This work |
| R3A4 | BAC containing *S. rimosus* chromosome fragment with OTC cluster | This work |
| R3A4::OTC | Derivate of R3A4 with replaced OTC cluster with antibiotic cassette | This work |
| patt-sHyg-Ery-oriT | Ap^R^; containing hygromycin and erythromycin resistance flanked by P-GG and B-CC used for IMES technique | This work |
| pUWLint31 | Ap^R^, Ts^R^; Streptomyces vector for the expression of ΦC31 integrase (*int* gene) from high copy number vector pUWL101 | (7) |

Ap^R^, apramycin resistant; Kan^R^, kanamycin resistant; Am^R^, ampicillin resistant; Erm^R^, erythromycin reistant; Ts^R^, thiostrepton resistant


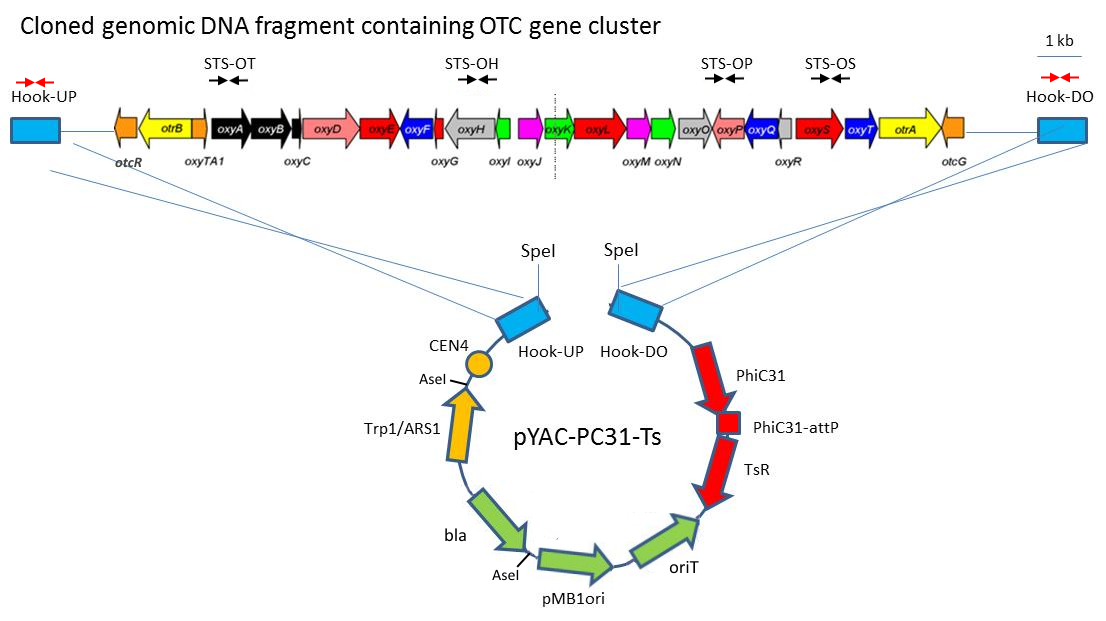


**Figure S1.** Schematic presentation of the plasmid containing the entire *otc* gene cluster, obtained via a single-step transformation-associated recombination cloning approach. The homologies used to clone the entire gene cluster are located externally at both ends of the *otc* gene cluster, and are labeled in blue. The *S. cerevisiae*/ *E. coli*/ *Streptomyces* shuttle vector is approximately 7400 bp long, each homology for homologous recombination is approximately 1 kb long, and *otc* cluster is approximately 28 kb long. The black arrows indicate primers used to screen for the correct plasmid. The red arrows above left and right homology, Hook-Up and Hook-Do, respectively, indicate primers used to create Southern blotting probes, which were used for *otc* cluster identification in Figure 4.


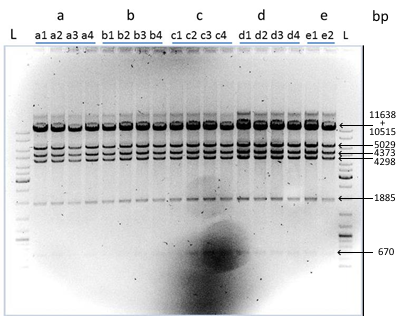


**Figure S2:** *Sac*I restriction analysis of individual plasmid constructs that were assumed to contain the entire *otc* gene cluster. The restriction pattern of pYAC-ΦC31-Ts-OTC (38408 bp) digested with *Sac*I, demonstrated seven bands of 11638 bp and 10515 bp (these two appear as one band on the agarose gel), 5029 bp, 4373 bp, 4298 bp, 1885 bp, and 670 bp clones. (**a-e**) Transformants obtained from genomic DNA of the individual yeast transformants previously selected by colony PCR. (**a1-a4**) Individual plasmid rescue clones from *E. coli*.


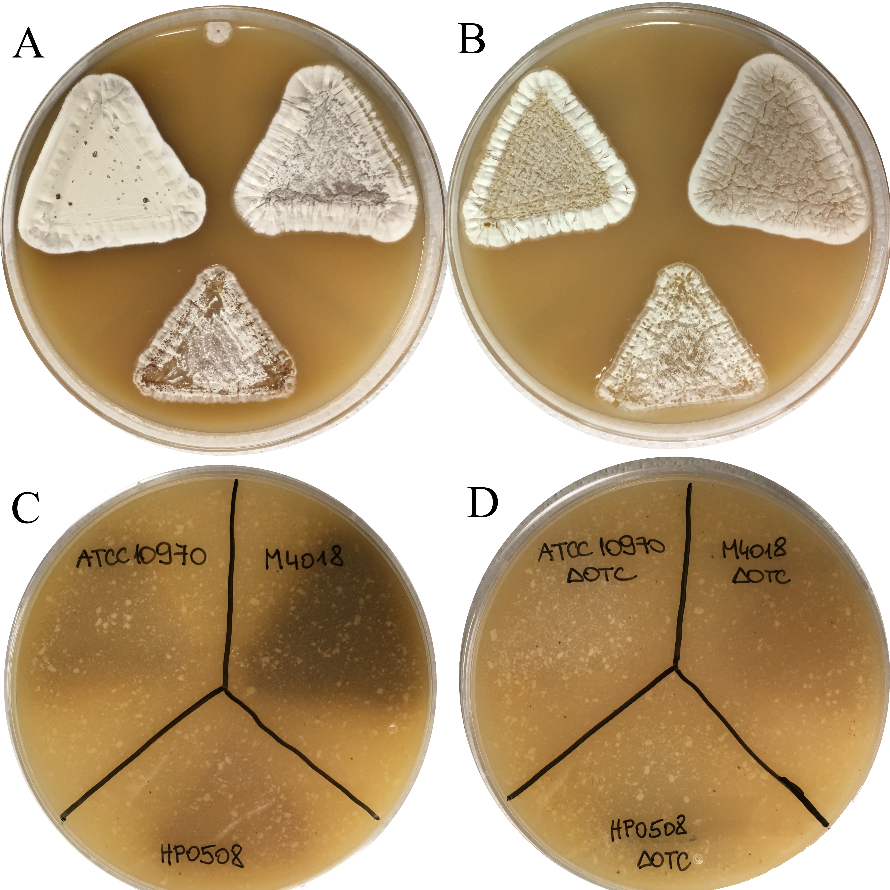


**Figure S3:** Morphological properties of the wild-type *S. rimosus* ATCC 10970 strain, the M4018 medium OTC-producing strain, and the HP0508 high OTC-producing strain, and of the same strains with an entire *otc* cluster deleted. (**A, B**) Upper of the MS plates, showing *S. rimosus* ATCC 10970, M4018, and HP0508 (**A**), and showing the *S. rimosus* ΔOTC mutants (**B**). (**C, D**) Bottom of MS plates, showing of *S. rimosus* ATCC 10970, M4018 and HP0508 (**C**), and showing the *S. rimosus* ΔOTC mutants (**D**). All three of the strains show slightly darker brown mycelia, as observed at the bottom of the plate. The colour has been correlated with OTC production; however, the pigment is of an unknown source, and its intensity does not directly correspond to the OTC titer, as the M4018 strain, which is a medium OTC-producer, shows the most intense dark color. The dark pigmentation disappears following *otc* gene cluster deletion. All three of the strains produce white spores on top of the mycelia; however, sporulation is reduced with higher OTC production, and HP0508 shows the lowest number of spores, indicating non-homogenous growth of the spores (Figure S3A, B).


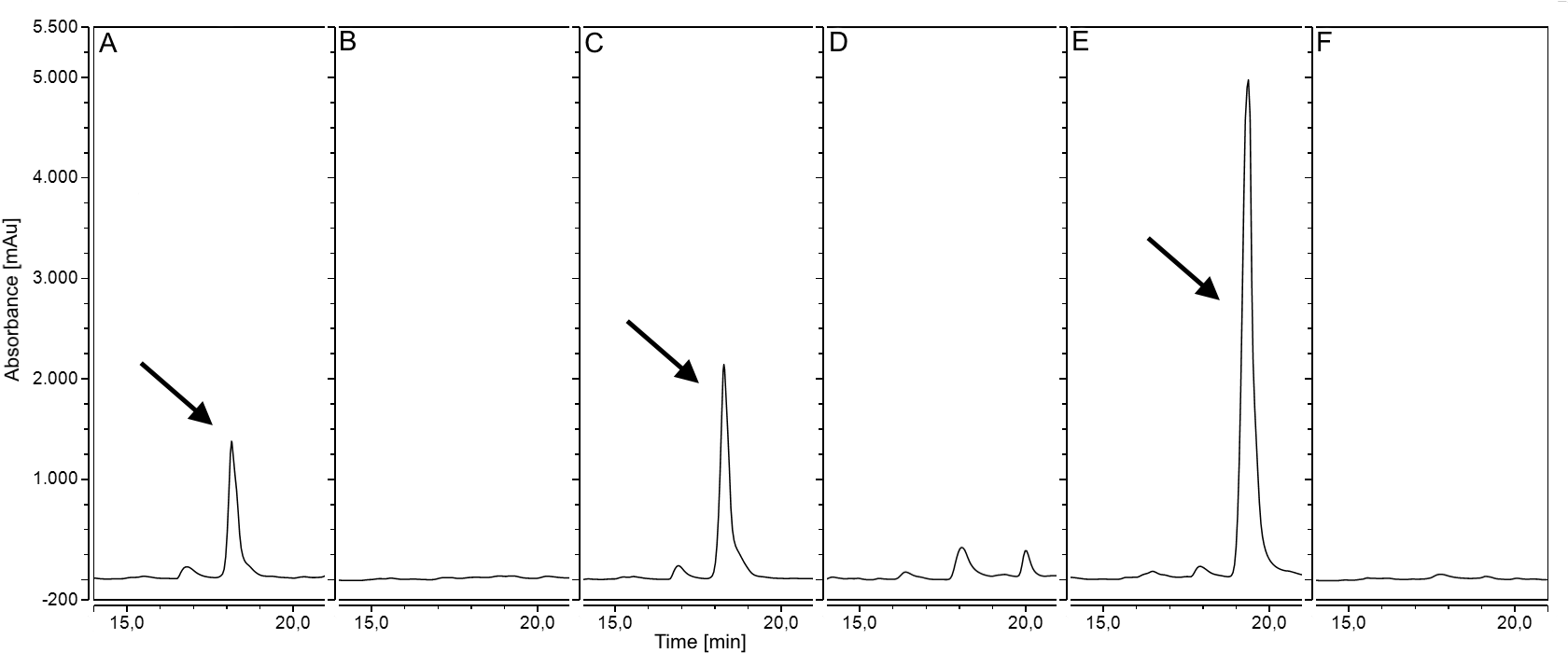


**Figure S4:** HPLC analysis of the OTC produced by different *S. rimosus* strains and their respective OTC deletion mutants. (**A**) ATCC 10970. (**B**) ATCC 10970ΔOTC. (**C**) M4018. (**D**) 15883S. (**E**) HP0508. (**F**) HP0508ΔOTC. The arrows indicate OTC with a retention time of approximately 18.2 min. OTC was quantified using a standard and calculated from the area under curve.


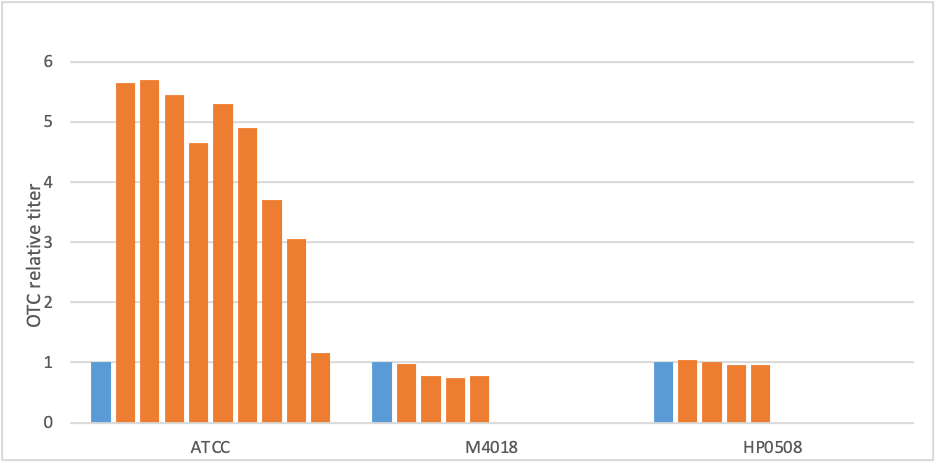


**Figure S5:** OTC titer following complementation of the *S. rimosus* ATCC10970 ΔOTC, 15883S, and HP0508 ΔOTC deletion mutants with an entire *otc* gene cluster. Blue bars, relative OTC titers of the parent *S. rimosus* strains: ATCC 10970, M4018, HP0508. Orange bars, relative OTC titers of the complemented ΔOTC mutants.


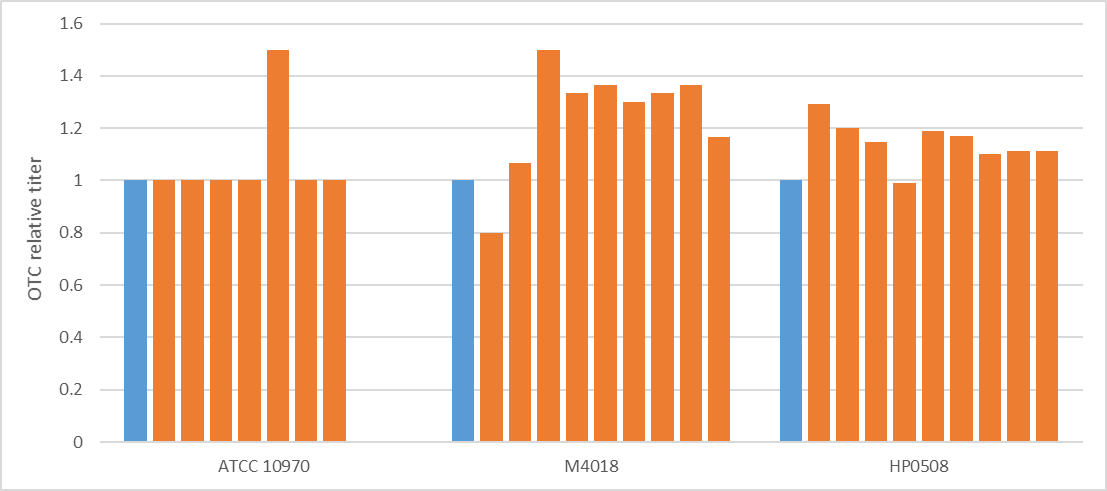


**Figure S6:** OTC titers from the independent transformants carrying the pYAC-ΦC31-Ts-h control plasmid (i.e., without the *otc* cluster) in *S. rimosus* ATCC 10970, M4018, and HP0508. Blue bars, relative OTC titers of the parent *S. rimosus* strains: ATCC 10970, M4018, HP0508. Orange bars, relative OTC titers of the independent pYAC-ΦC31-Ts-h transformants. The significance of the variability was tested with two-tailed Mann-Whitney tests. No significant variability was detected for the ATCC 10970 strain transformed with the control plasmid (P >0.05), while significant variability was detected for the M4018 and HP0508 transformants with the control (P <0.05).


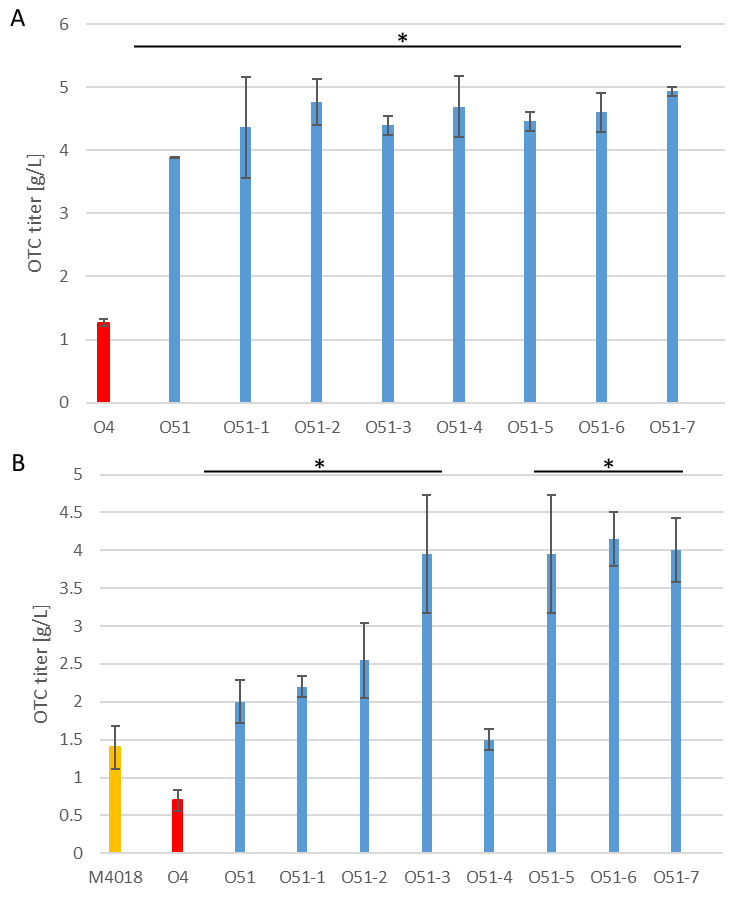
**Figure S7:** OTC titers of the initial O51 transformant and of seven colonies derived from the O51 colony. M4018 parental strain and pYAC-ΦC31-Ts-h O4 were taken as the control strains. (**A**) Vegetative medium with thiostrepton. (**B**) Vegetative medium without thiostrepton. The titers were measured after 5 days of fermentation in 5 mL GOTC production medium. * P <0.05 (vs. control O4 parental strain; two-tailed Mann-Whitney test).


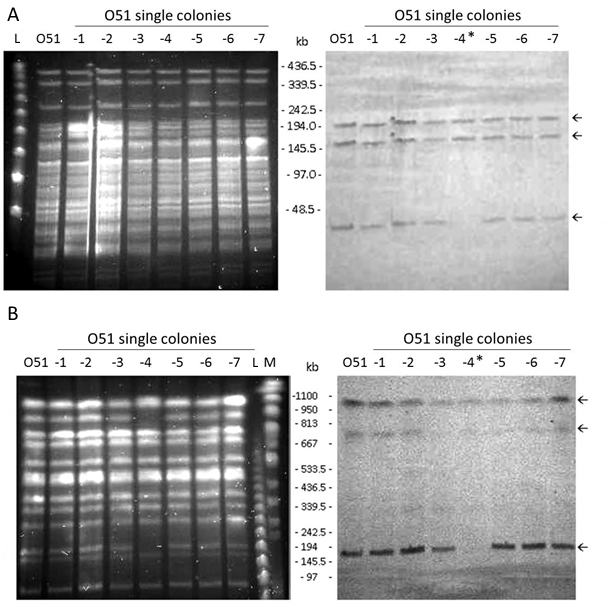


**Figure S8:** The stabilities of the integrated copies of the *otc* gene clusters in the initial O51 transformant and for its derived single colonies (O51-1-7) after vegetative growth in TSB medium without thiostrepton (**A**) *Xho*I digestion. (**B**) *Ase*I digestion. L, Lambda concatamers, M, *S. cerevisiae* chromosomes. Right: Southern blotting of the PFGE gels, hybridized with probes generated from external homologs of pYAC ΦC31-Ts-h. Arrows indicate the *otc* cluster that is present in the band. Asterisk indicates colony O51-4, which lost one copy of the *otc* cluser during the growth in TSB medium.


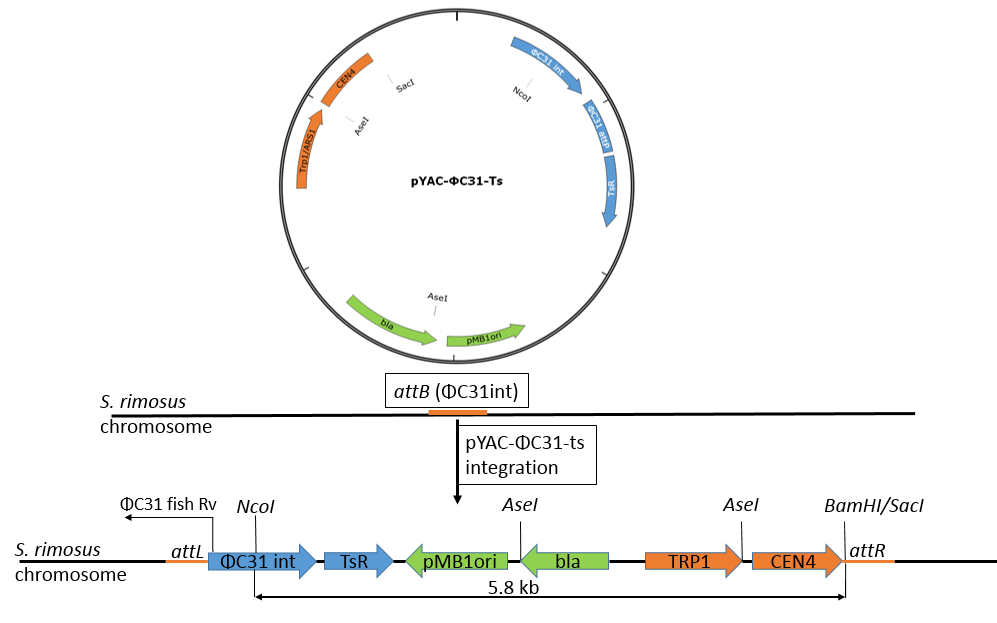


**Figure S9:** Schematic representation of plasmid pYAC-ΦC31-Ts and its integration into the *S. rimosus* chromosome.


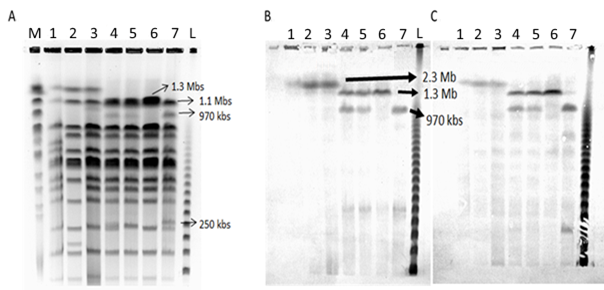


**Figure S10:** (**A**) Pulse-field gel electrophoresis (PFGE) analysis of chromosomal DNA digested with *Ase*I. (**B)** Membrane treated with a probe generated with the Fw P1 and Rv P1 primers (Table S2). (**C**) Membrane treated with a probe generated with the Fw P2 and Rv P2 primers. Lanes: M, Saccharomyces cerevisiae chromosome marker; 1, ATCC 10970; 2, 15883S; 3, M4018; 4. 5. 6. 7, S. rimosus: YAC transformants. L: Lambda PFGE ladder.


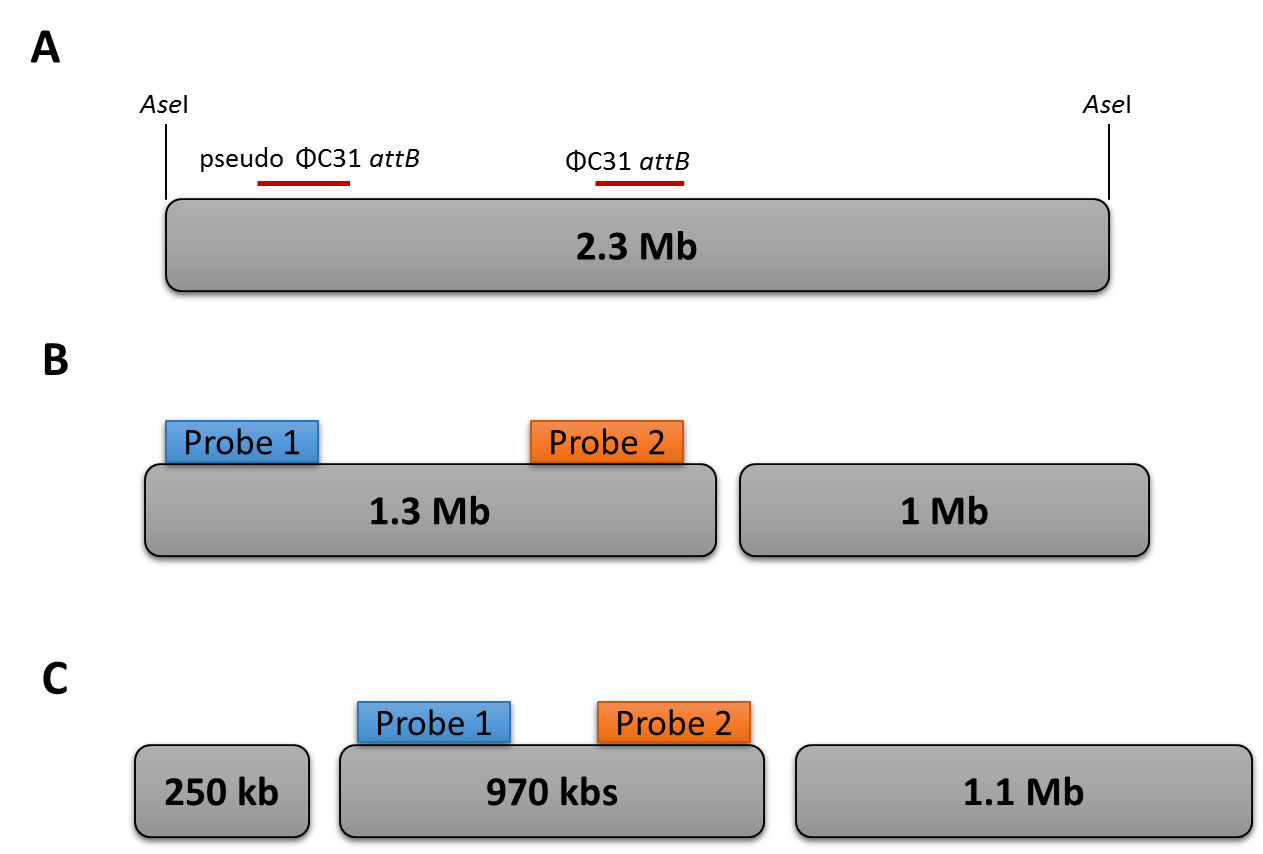


**Figure S11:** Possible integration events of plasmid pYAC-ΦC31-Ts. (**A**) The largest fragment of the *S rimosus* M4018 genome (2.3 Mb), after restriction with an *Ase*I restriction nuclease. This carries both perfect and pseudo ΦC31 *attB* sites. (**B**) Single integration event at the perfect ΦC31 *attB*. In Figure S10, this corresponds to transformants in lane 6 and a subpopulation of transformants in lanes 4 and 5. (**C**) Double integration event. In Figure S10, this corresponds to transformants in lane 7 and a subpopulation of transformants in lanes 4 and 5. Plasmid pYAC-ΦC31-Ts contains two *AseI* restriction sites; however, as they are close together (approximately 1 kb), they cannot be separated by PFGE, and are thus presented as one *AseI* site.


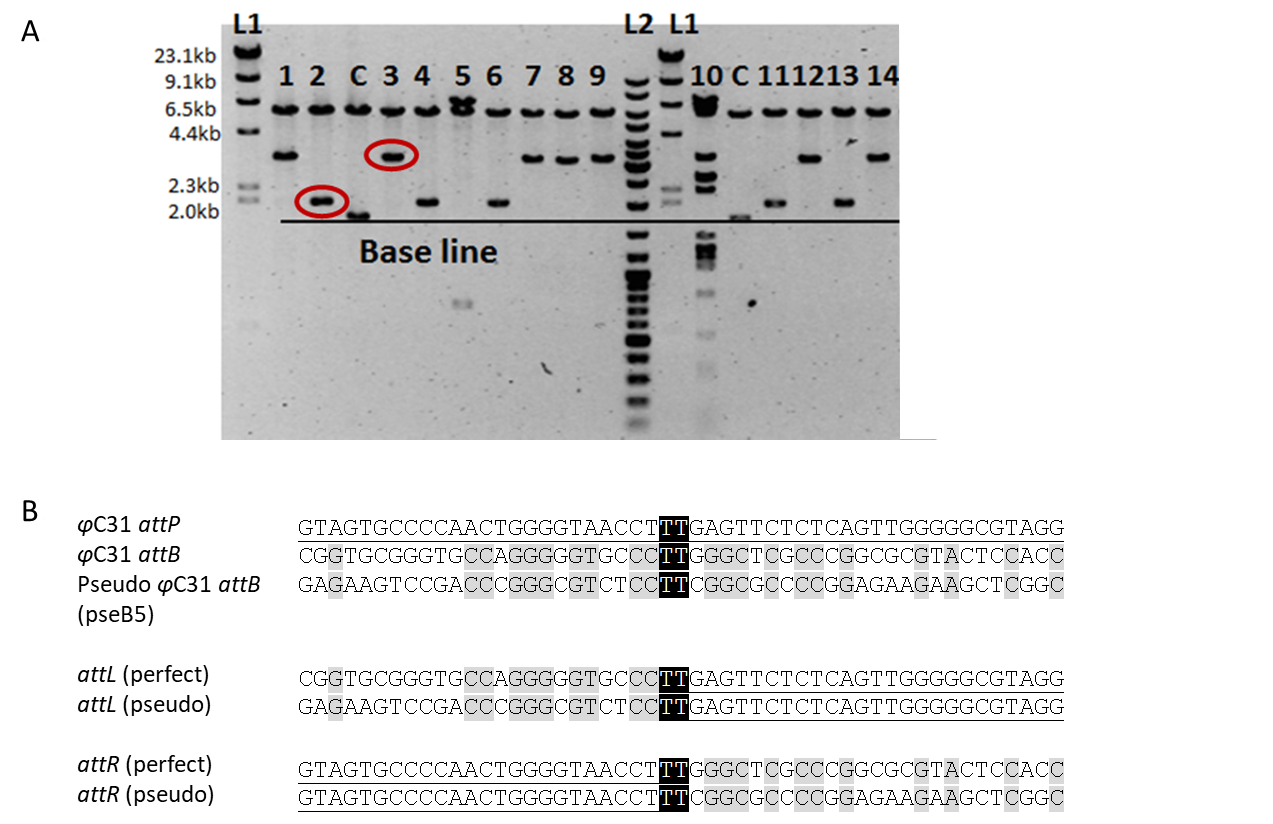


**Figure S12:** (**A**) Restriction patterns of the pYAC-ΦC31-Ts plasmid and rescued plasmids generated with *BamH*I*/Nco*I. pYAC-ΦC31-Ts vector (7.5 kb) digested with *BamH*I*/Nco*I shows titers with two bands of 1.7 kb and 5.8 kb. L1, Lambda DNA/HindIII ladder; L2, 1 kb gene ruler; C, pYAC-ΦC31-Ts *BamH*I*/Nco*I; 1-14, rescued plasmids digested with *BamH*I*/Nco*I. All of the plasmids show the corresponding 5.8 kb band (core structure of pYAC-ΦC31-Ts); the rescued plasmids showed two different patterns compared to the base line in pYAC-ΦC31-Ts vector. (**B**) Sequences of the attachment sites recognized by ΦC31 *int/attP* recombinase in the *S. rimosus* genome. Comparison of *attP*, *attB*, and pseudo *attB* sites before integration and modification of *attB* (*attL* and *attR*) sites after integration. The *attP* sequence is underlined, conserved nucleotides of the *attB* sites are highlighted, and the core region 5´TT where cross-over occurs is indicated inside the black box.


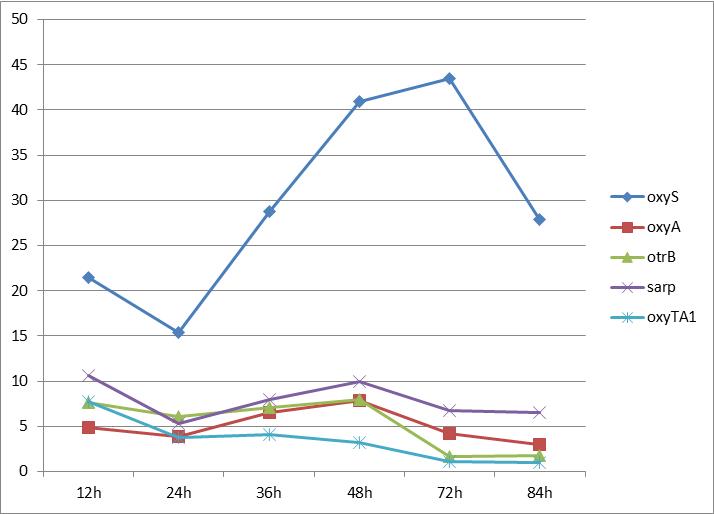


**Figure S13:** Ratios of the selected gene expression in the *S. rimosus* O51 transformant, compared to the M4018 parent strain.


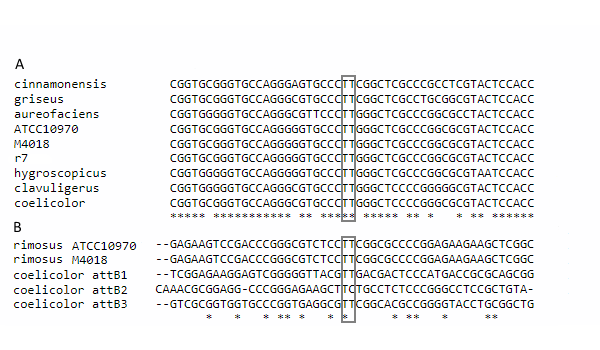


**Figure S14:** (**A**) Alignment of DNA sequences corresponding to the perfect ΦC31 *attB* sites identified in the different *Streptomyces* strains: *Streptomyces cinnamonensis* (cinnamonensis); *Streptomyces griseus* (griseus); *Kitasatospora aureofaciens* (aureofaciens); *Streptomyces rimosus* ATCC 10970 (ATCC10970); *Streptomyces rimosus* M4018 (M4018); *Streptomyces rimosus* R7 (r7); *Streptomyces hygroscopicus* (hygroscopicus); *Streptomyces* *clavuligerus* (clavuligerus); *Streptomyces coelicolor* (coelicolor). (**B**) Alignment of the DNA sequences of the pseudo ΦC31 *attB* sites identified in the different *Streptomyces* *rimosus* and *Streptomyces coelicolor* strains. The core TT signature where the crossover occurs is highlighted inside the gray box. Nucleotides sharing identity are denoted with asterisks.


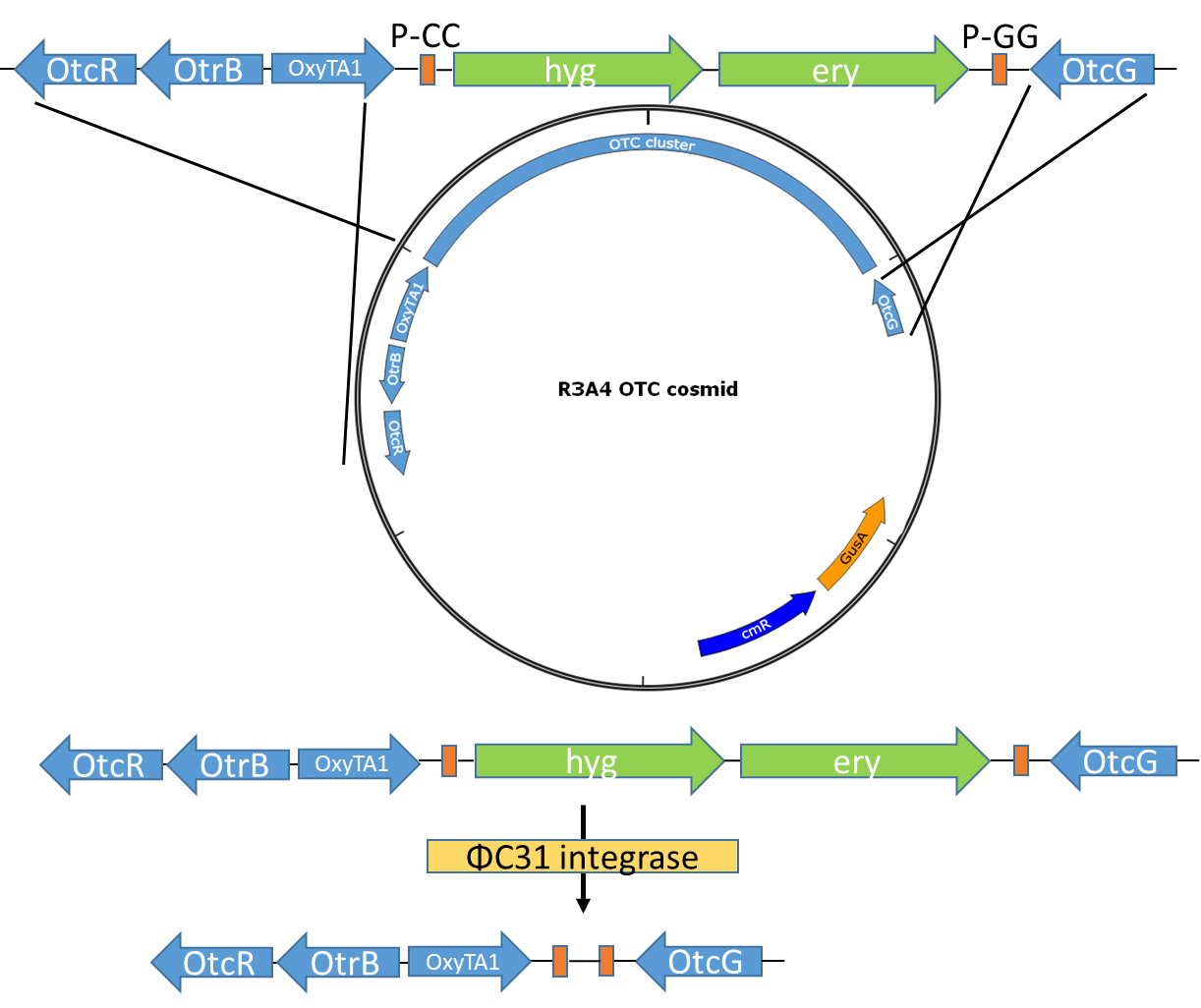


**Figure S15:** Schematic presentation of the *otc* cluster deletion in *S. rimosus* ATCC 10970 using an iterative marker excision system.

**3 References**

1. Sobin BA, Finlay AC, Kane JH. Terramycin and its production. United States Patent and Trademark Office. 466; 1950.

2. Rhodes PM, Hunter IS, Friend EJ, Warren M. Recombinant DNA methods for the oxytetracycline producer *Streptomyces rimosus*. Biochem Soc Trans. 1984;12(4):586–7.

3. McDowall KJ, Thamchaipenet A, Hunter IS. Phosphate control of oxytetracycline production by *Streptomyces rimosus* is at the level of transcription from promoters overlapped by tandem repeats similar to those of the DNA-binding sites of the OmpR family. J Bacteriol. 1999;181(10):3025–32.

4. Sambrook J, Russell DW. Molecular Cloning. A Laboratory Manual. 3rd ed. Cold Spring Harbor, N.Y.: Cold Spring Harbor Laboratory Press; 2001. 2344 H2-572.8 21British Library DSC m01/18384 T.

5. Flett F, Mersinias V, Smith CP. High efficiency intergeneric conjugal transfer of plasmid DNA from Escherichia coli to methyl DNA-restricting streptomycetes. FEMS Microbiol Lett. 1997;155(2):223–9.

6. MacNeil DJ, Gewain KM, Ruby CL, Dezeny G, Gibbons PH, MacNeil T. Analysis of *Streptomyces avermitilis* genes required for avermectin biosynthesis utilizing a novel integration vector. Gene. 1992;111(1):61–8.

7. Myronovskyi M, Rosenkränzer B, Luzhetskyy A. Iterative marker excision system. Appl Microbiol Biotechnol. 2014;98(10):4557–70.
